# Glucose deprivation elicits phenotypic plasticity via ZEB1-mediated expression of NNMT

**DOI:** 10.18632/oncotarget.15429

**Published:** 2017-02-17

**Authors:** Justyna Kanska, Paul-Joseph P. Aspuria, Barbie Taylor-Harding, Lindsay Spurka, Vincent Funari, Sandra Orsulic, Beth Y. Karlan, W. Ruprecht Wiedemeyer

**Affiliations:** ^1^ Women's Cancer Program at the Samuel Oschin Cancer Institute, Cedars-Sinai Medical Center, Los Angeles, CA 90048, USA; ^2^ Genomics Core, Cedars-Sinai Medical Center, Los Angeles, CA 90048, USA; ^3^ Department of Obstetrics and Gynecology, David Geffen School of Medicine, University of California, Los Angeles, CA 90048, USA

**Keywords:** nicotinamide N-methyltransferase, ovarian cancer, chronic nutritional stress, mesenchymal gene expression, cancer metabolism

## Abstract

Glucose is considered the primary energy source for all cells, and some cancers are addicted to glucose. Here, we investigated the functional consequences of chronic glucose deprivation in serous ovarian cancer cells. We found that cells resistant to glucose starvation (glucose-restricted cells) demonstrated increased metabolic plasticity that was dependent on *NNMT* (Nicotinamide N-methyltransferase) expression. We further show that ZEB1 induced *NNMT*, rendered cells resistant to glucose deprivation and recapitulated metabolic adaptations and mesenchymal gene expression observed in glucose-restricted cells. NNMT depletion reversed metabolic plasticity in glucose-restricted cells and prevented *de novo* formation of glucose-restricted colonies. In addition to its role in glucose independence, we found that NNMT was required for other ZEB1-induced phenotypes, such as increased migration. NNMT protein levels were also elevated in metastatic and recurrent tumors compared to matched primary carcinomas, while normal ovary and fallopian tube tissue had no detectable NNMT expression. Our studies define a novel ZEB1/NNMT signaling axis, which elicits mesenchymal gene expression, as well as phenotypic and metabolic plasticity in ovarian cancer cells upon chronic glucose starvation. Understanding the causes of cancer cell plasticity is crucial for the development of therapeutic strategies to counter intratumoral heterogeneity, acquired drug resistance and recurrence in high-grade serous ovarian cancer (HGSC).

## INTRODUCTION

Cellular energy metabolism is one of the first processes altered during neoplastic transformation and its deregulation is one of the emerging hallmarks of cancer [1, 2]. Rapidly proliferating cancer cells increase glucose uptake and undergo aerobic glycolysis (“Warburg effect” [1, 3, 4]) in order to meet their increased metabolic demands for the biosynthesis of nucleolipids, lipids, amino acids and NADPH [5]. This phenomenon can be visualized by positron emission tomography (PET), which utilizes glucose analogs as reporters [6]. PET imaging is currently an indispensable tool in diagnostic oncology for monitoring neoplastic transformation in patients [7]. Interestingly, there are “PET”-negative tumors, suggesting that some cancers have a low glucose uptake [8]. These tumors could be less dependent on glycolysis (i.e. have a low glycolytic activity), too small for detection, or unable to access the glucose supply as a result of a poorly developed vasculature [8, 9]. Indeed, the intratumoral glucose concentrations in some solid tumors, such as breast, colon and prostate cancers, appear to be lower than in normal cells of the same tissue sites [10]. Also, there are profound inter- and intratumoral differences in the tumor vasculature [11–13] and poorly vascularized regions of the tumor may have restricted glucose access, causing a heterogeneous intratumoral glucose distribution. This is partly due to the limited distance to which oxygen and nutrients can diffuse from tumor blood vessels [14]. Cells residing in close proximity to blood vessels are highly oxygenated and well-nourished (glucose levels >>2.5 mM), while those residing farther away are hypoxic and have low glucose levels (< 2.5 mM) [14]. Furthermore, a study of invasive ovarian carcinomas showed that tumor cell-lined vasculature was present only in 30% of tumors (23 out of 77 patients) [15], suggesting that up to two thirds of all ovarian cancers may have regions with underdeveloped vasculature. In addition, the use of anti-angiogenic agents, such as bevacizumab, which was recently FDA-approved for recurrent high-grade serous ovarian cancer (HGSC), impairs tumor vasculature [16–21]. Since the main role of bevacizumab is to deprive the tumor of its blood supply in order to induce cancer cell starvation and apoptosis, bevacizumab-treated cancers are exposed to nutritional stress, such as glucose deprivation. Taken together, these data suggest that some regions of advanced solid tumors, including HGSC, are deprived of glucose. Cancers forced to adapt to a nutrient-deprived environment may select for highly metabolically plastic cells or rewire their metabolism in order to survive. Thus, it is crucial to understand the consequences of adaptation to nutritional stress as they may substantially influence ovarian cancer phenotype during cancer expansion or following anti-angiogenic therapy. A better understanding of metabolic events influencing tumor behavior may inform the design and outcome of novel treatment strategies. To date, a study on phenotypic consequences of chronic glucose withdrawal on ovarian cancer cells has not been performed.

Previous studies have focused on the effects of acute glucose deprivation on signaling pathways that govern cellular survival or death upon glucose withdrawal [22–24]. Short-term glucose starvation in ovarian cancer cells was shown to upregulate *SLC2A1* and *G6PD* mRNA and protein levels [25]. SLC2A1 (GLUT1) is a constitutive, high affinity glucose transporter with additional substrate specificity for transporting various pentoses and hexoses [26, 27]. G6PD (Glucose-6-phosphate dehydrogenase) is a rate-limiting enzyme of the Pentose Phosphate Pathway (PPP), whose main function is to generate reducing agents (NADPH) and pentose phosphates for nucleic acids and lipid synthesis [28–30]. Pasto *et al*. also showed that glucose deprivation enriched for CD44^+^ CD117^+^ ovarian cancer cells with cancer stem cell (CSC)-like characteristics, which exhibit higher oxidative phosphorylation (OXPHOS), elevated reactive oxygen species (ROS) levels and PPP activity [25]. However, little is known about the phenotypic consequences of chronic glucose deprivation in cells that survive the initial shock of glucose withdrawal. Here, we sought to uncover the genetic, transcriptional and metabolic adaptations in serous epithelial ovarian cancer cells in response to chronic glucose starvation. We found that resistance to glucose deprivation correlates with increased expression of specific metabolic genes that include *G6PD, SLC2A1*, as well as *NNMT* (Nicotinamide N-methyltransferase), the function of which in this context was previously unknown. Our studies reveal that NNMT is required for glucose independence and enables glucose-deprived cells to utilize a number of alternative substrates as energy sources in the absence of sufficient glucose levels. We further show that NNMT is induced as part of a ZEB1-mediated mesenchymal gene expression program, which determines the metabolic and phenotypic plasticity in glucose-restricted cells. While ZEB1 is a known inducer of epithelial-to-mesenchymal transition (EMT), we find that EMT is not required for glucose independence. Rather, our data suggest that NNMT requirement in glucose-restricted cells selects for ZEB1 expression, which may in turn result in partial or full EMT and thus enhance cancer cell plasticity. Therefore, nutritional stress may contribute to intratumoral heterogeneity, a hallmark feature of HGSC that is considered to play a role in its high rate of recurrence and poor overall survival [31–35].

## RESULTS

### Glucose deprivation induces *NNMT* expression

**Figure 1:**
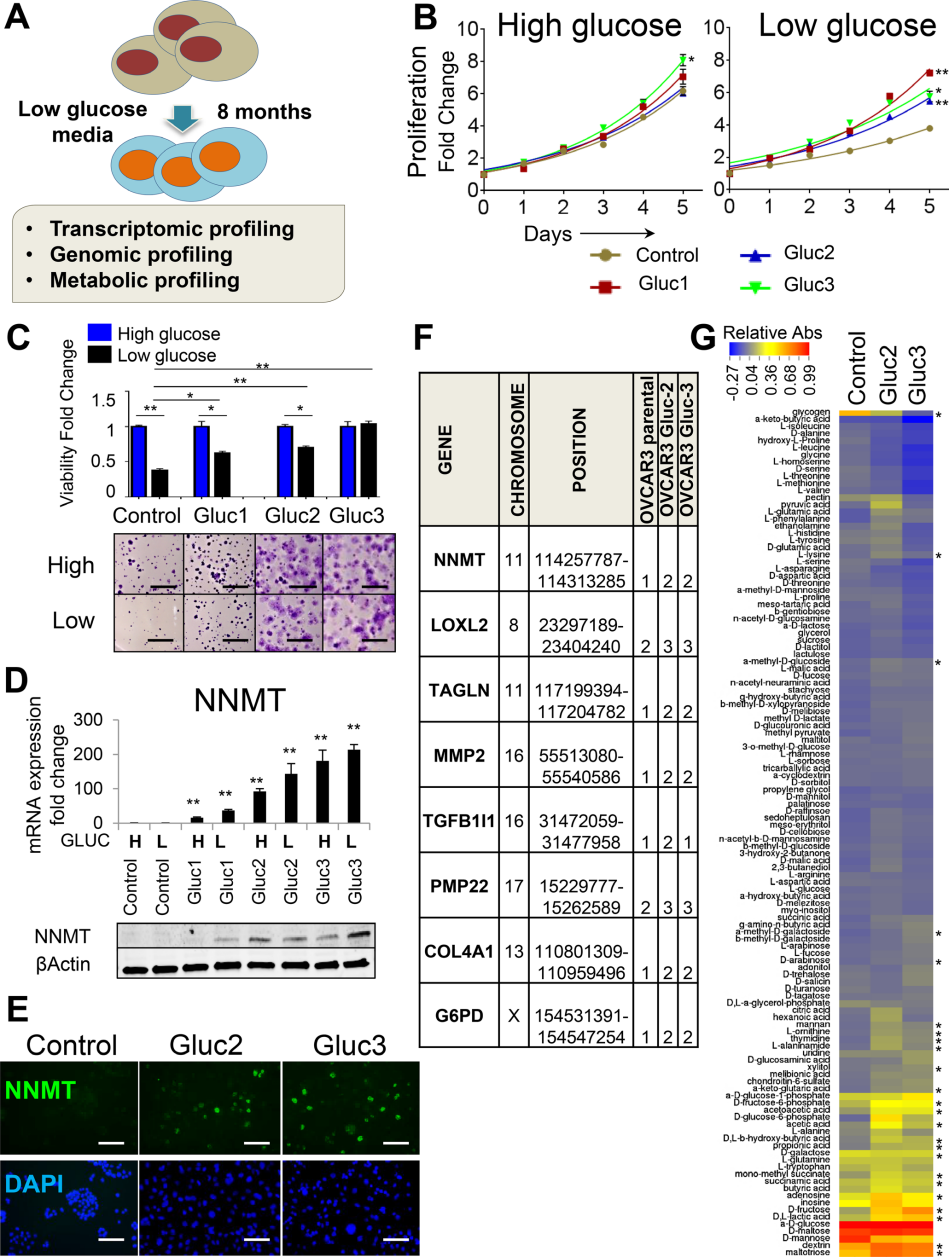
Glucose deprivation induces NNMT expression in OVCAR3 cells (**A**) Study layout depicting the generation and characterization of glucose-restricted sublines derived from OVCAR3 cell line. (**B**) Glucose-restricted OVCAR3 sublines sustain high proliferative capacity in low glucose levels in normal seeding density conditions, whereas proliferation of control cells is diminished. Differences in total cell number (measured by a luminometric viability assay) were evaluated on day 5 and marked with asterisks if statistically significant. (**C**) Glucose-restricted OVCAR3 sublines show increased viability in low glucose conditions compared to control cells. Cells were seeded at low density and allowed to expand for 18 d in DMEM with low glucose (black bars) or high glucose (blue bars) before they were stained with crystal violet. The bar graph presents relative viability of each individual subline after 18 d. Pictures below the graph show general difference in the appearance of the colonies. Scale bar: 5 mm. (**D**) NNMT RNA and protein levels are elevated in glucose-restricted OVCAR3 sublines (Gluc) and remain high even after 7 d culture in high glucose DMEM (H). Exposure of control cells to short-term (2 d) culture in DMEM with low glucose (L) levels did not induce NNMT expression. All cells were cultured in normal-seeding density conditions. (**E**) NNMT immunofluorescence shows increased number of NNMT-expressing cells in glucose-restricted OVCAR3 sublines (Gluc-2 and Gluc-3) and no detectable NNMT staining in the control. Scale bar: 50 μm. (**F**) Microarray-based CGH analysis of glucose-restricted OVCAR3 sublines reveals genomic gains in chromosomal regions harboring mesenchymal (*LOXL2*, *TAGLN*, *MMP2, COL4A1, TGFB1I1*) and metabolic genes (*NNMT*, *G6PD*). Copy number values are as follows: 0: loss, 1: deletion, 2: two copies (diploid), 3: three copies, 4: 4+ copies (amplification). (**G**) Biolog Microarray results show that glucose-restricted OVCAR3 sublines in the absence of glucose efficiently utilize additional energy sources in contrast to the control cells. The heatmap illustrates relative substrate utilization, which is defined by an absorbance value normalized by deducting the negative control absorbance (uncoated well) from the absorbance value of a well coated with a given substrate, and dividing it by the average of positive control (wells coated with α-D-glucose). Substrates differentially utilized and showing statistical significance (*P* < 0.05) in both Gluc-2 and Guc-3 are marked with asterisks. For all figure panels, statistical calculations were performed using a two-tailed Student's *t*-test (* 0.001 < *P* <0.05; **P < 0.001). All cells were harvested at ≤ 80% confluency.

In order to assess the impact of glucose deprivation in epithelial ovarian cancer cell lines, we serially cultured OVCAR3 cells in DMEM without added glucose. Due to trace amounts of glucose in fetal bovine serum (FBS), cells cultured in glucose-free DMEM with 10% FBS are exposed to extremely low levels of glucose (∼0.125 g/l ≈ 0.69 mM), similar to glucose levels observed in hypoxic and necrotic regions of solid cancers (< 2.5 mM) [14] (Figure 1A). Control cells were continuously passaged in regular DMEM containing 4.5 g/l glucose (25 mM, hereafter referred to as high glucose levels). After eight months, three independently derived glucose-restricted populations of cells (OVCAR3 Gluc-1–3 sublines) were compared to control cells in the presence of high and low glucose levels. In regular seeding density conditions in high glucose DMEM, glucose-restricted OVCAR3 sublines proliferated at similar rates as control cells; however, proliferation of control cells was drastically diminished in low glucose conditions, in which glucose-restricted cells were not affected (Figure 1B). During prolonged (18 d) culturing in low density conditions, glucose-restricted OVCAR3 sublines maintained their capacity to proliferate and form viable colonies, whereas viability of control cells was drastically impaired. Specifically, the number of viable Gluc-3 cells was virtually indistinguishable between high and low glucose conditions, while the number of viable control cells was reduced at least two-fold in low glucose DMEM (Figure 1C). This more stringent assay also revealed phenotypic differences between the three sublines, where the OVCAR3 Gluc-1 subline had an intermediate phenotype between glucose deprivation-sensitive control OVCAR3 cells and fully adapted to glucose withdrawal OVCAR3 Gluc-3 cells (Figure 1C).

To determine genome-wide transcriptional changes underlying glucose independence in OVCAR3 cells, we performed RNA sequencing (RNAseq) on glucose-restricted cells (OVCAR3 Gluc-2 and Gluc-3) and control OVCAR3 cells (Supplementary Table 1). We have found 613 genes differentially expressed between both Gluc-2 and Gluc-3 sublines and control cells, out of which 243 genes were upregulated and 370 downregulated. Expression of those genes was altered at least two-fold in both sublines (*P* < 0.05, *q* < 0.2). We have identified Nicotinamide N-methyltransferase, *NNMT*, as the most upregulated gene in glucose-restricted OVCAR3 cells (> 140-fold increase in both Gluc-2 and Gluc-3 in two biological replicates, *P* = 0.002 for Gluc-2; *P* = 0.0004 for Gluc-3, Supplementary Table 1, Figure 1D). RNA and protein levels of *NNMT* correlated with the extent of adaptation to glucose deprivation, with no detectable *NNMT* expression in control OVCAR3, mild induction in OVCAR3 Gluc-1, and highest *NNMT* expression in OVCAR3 Gluc-2 and Gluc-3 sublines (Figure 1D). This expression pattern was confirmed by immunofluorescence (IF), which detected robust NNMT protein staining in OVCAR3 Gluc-2 and Gluc-3 cells but not in control OVCAR3 (Figure 1E). Staining intensity varied considerably between individual glucose-restricted cells. Since OVCAR3 Gluc-2 and Gluc-3 sublines demonstrated the best adaptation to glucose starvation, we prioritized Gluc-2 and Gluc-3 cells for further genomic and metabolic profiling. Using array-based Comparative Genomic Hybridization (aCGH) we found genomic copy number gain of *NNMT* in both OVCAR3 Gluc-2 and Gluc-3 cells compared to control (Figure 1F). Additionally, several other genomic regions showed copy number alterations in glucose-restricted cells, including the chromosomal region harboring *G6PD* gene (Figure 1F and Supplementary Table 2). In line with a published study on ovarian cancer stem cells that are more resistant to glucose deprivation [25], glucose-restricted OVCAR3 cells induced *G6PD* and *SLC2A1* expression at the transcriptional level (Supplementary Table 1 and Supplementary Figure 1A). In summary, we identified robust upregulation of the methyl transferase gene, *NNMT*, in glucose-restricted ovarian cancer cells, along with upregulation of genes that were previously described in the context of glucose starvation, such as *G6PD* and *SLC2A1*.

### Glucose-restricted OVCAR3 cells acquire distinct metabolic adaptations

To determine if glucose-restricted ovarian cancer cell lines rewire their metabolic pathways in order to compensate for the lack of glucose, we analyzed their ability to utilize alternative metabolites as energy sources in the absence of glucose. For this purpose, we used Biolog Phenotype Microarrays, in which wells are individually coated with different carbon and nitrogen substrates that can potentially serve as energy sources in low glucose levels (Figure 1G). Compared to control OVCAR3 cells, the OVCAR3 Gluc-2 and Gluc-3 sublines acquired distinct patterns of substrate utilization, with the most striking differences seen in their utilization of various sugars and methylated substrates, and with reduced utilization of many amino acids (Figure 1G). We prioritized substrates, which utilization was statistically significant between glucose-restricted cells (OVCAR3 Gluc-2 and Gluc-3) and control cells (Figure 1G, asterisks). We observed that in low glucose conditions, OVCAR3 Gluc-2 and Gluc-3 cells utilized other sugars, such as D-fructose, D-arabinose, mannan, maltotriose and dextrin more efficiently than control cells (Figure 1G), which may be a result of elevated *SLC2A1* expression (Supplementary Table 1 and Supplementary Figure 1A). In addition, glucose-restricted cells also utilized adenosine and D, L- lactic acid at significantly higher rates, both of which were previously shown to feed metabolism via gluconeogenesis or glycolysis [36, 37]. These results suggest that glucose-restricted OVCAR3 sublines can utilize other carbon sources to bypass the need for glucose.

In addition, D-glucose-6-phosphate, which is a G6PD substrate, was utilized at much higher levels in glucose-restricted OVCAR3 sublines compared to control cells (on average > 20-fold, *P* = 0.055) (Figure 1G). Along with upregulated *G6PD* expression, this result suggests that glucose-independent cells elevate Pentose Phosphate Pathway (PPP) activity, as previously described in glucose deprivation-resistant ovarian cancer stem cells [25]. In line with this study, we observed that glucose-restricted OVCAR3 Gluc-2 and Gluc-3 sublines had increased expression of ovarian cancer stem cell markers, such as *CD44* and *CD117* [35], as well as decreased expression of *CA125* (*MUC16*) [38], an ovarian cancer marker negatively correlated with stemness (Supplementary Table 1 and Supplementary Figure 1B).

Biolog Microarrays also showed that OVCAR3 Gluc sublines utilized more ketone bodies (acetoacetic acid and D, L-β-hydroxy-butyric acid; all *P* < 0.05) than control cells, suggesting that glucose-restricted sublines may rely more on ketosis, the process in which ketone bodies are used as a metabolic fuel. Interestingly, in contrast to control cells, glucose-restricted OVCAR3 cells utilized methylated compounds, such as α-methyl-D-galactoside, α-methyl-D-glucoside and mono-methyl succinate. Considering the role of NNMT in methylation reactions, the use of these methylated substrates may be a specific adaptation of glucose-restricted cells that may be linked to *NNMT* expression.

### *NNMT* and *G6PD* expression correlates with tolerance to glucose deprivation

**Figure 2:**
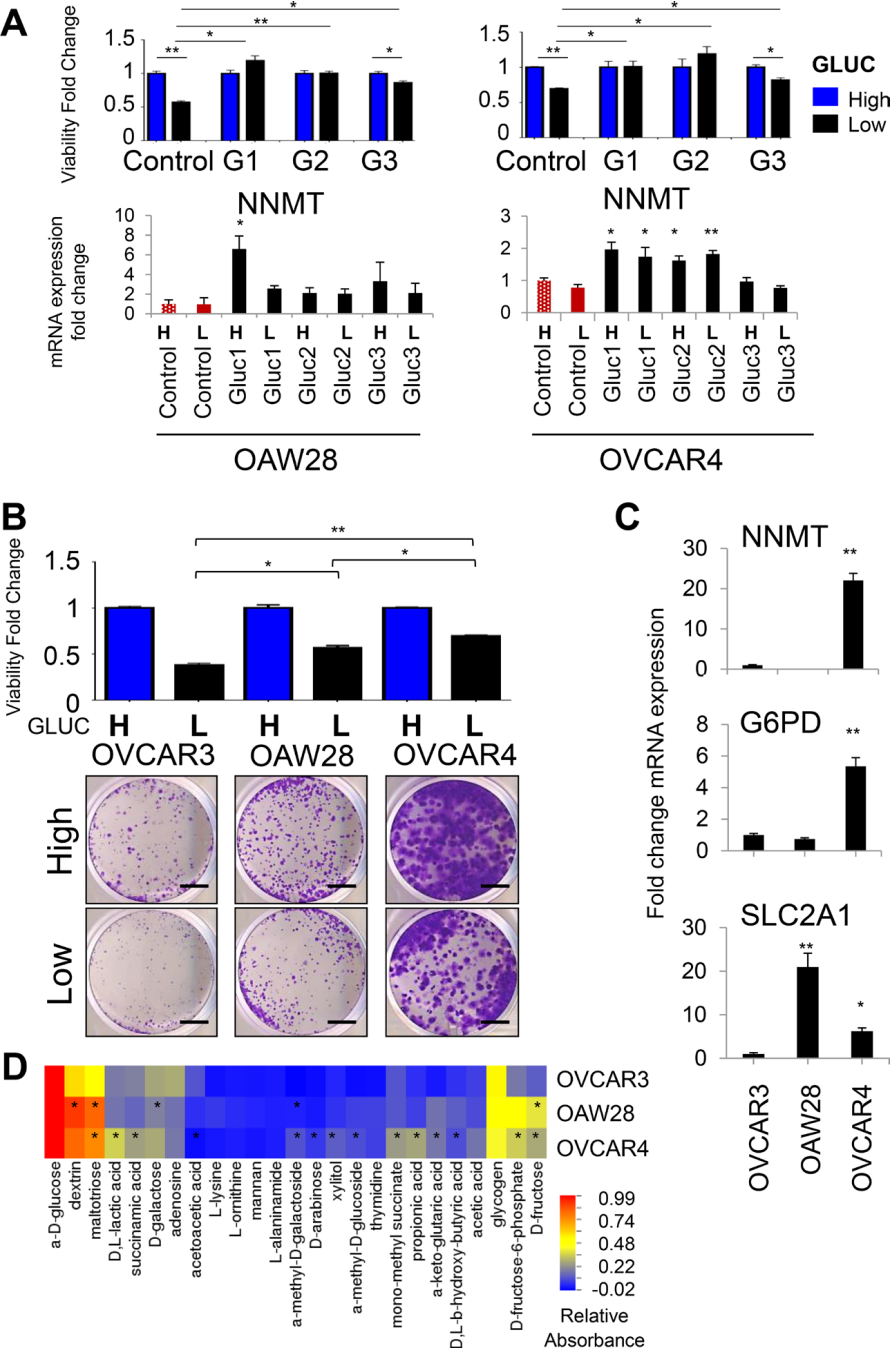
*NNMT* and *G6PD* expression correlates with tolerance to glucose deprivation in OVCAR4 and OAW28 cells (**A**) Cells were seeded at low density and allowed to expand for 18d in DMEM with low glucose (black bars) or in high glucose (blue bars). Using crystal violet staining we show that relative number of viable glucose-restricted OAW28 and OVCAR4 cells is similar in DMEM with low and high glucose levels, whereas viability of control cells is impaired upon glucose withdrawal. Graphs in the lower panel depict transcriptional changes in OAW28 and OVCAR4 glucose-restricted sublines determined by qRT-PCR. *NNMT* mRNA expression is consistently upregulated in all glucose-restricted sublines generated from OVCAR4 and OAW28 cell lines. *NNMT* mRNA levels remain largely unaffected upon 7 d-long release of glucose-restricted sublines (black bars) to high glucose DMEM (H). Similarly, 2 d-long culture of control cells (red bars) in low glucose DMEM (L) does not induce *NNMT* mRNA levels. (**B**) Parental OVCAR4 cells have a superior ability to expand in low density seeding conditions in low glucose levels, as manifested by the higher total number of viable cells in low glucose DMEM (black bars) compared to OAW28 and OVCAR3 cells. OAW28 cells have an intermediate phenotype. All cell lines were cultured for 18 d and treatment in low glucose levels was normalized to treatment in high glucose levels (blue bars) for the same cell line to account for the intrinsically different proliferation rates of all tested cell lines. Scale bar: 5 mm. (**C**) *NNMT* and *G6PD* mRNA expression is higher in parental OVCAR4 cells compared to OVCAR3 and OAW28 cell lines. Expression of *SLC2A1* (*GLUT1*) is highest in parental OAW28 cells. (**D**) Biolog Microarray results show that parental OVCAR4 cells utilize methylated substrates more efficiently than parental OVCAR3 cells. Parental OAW28 cells have an intermediate phenotype. Substrates differentially utilized between the given cell line and OVCAR3 cells are depicted with asterisk, if the difference is statistically significant (*P* < 0.05) in two independent experiments. For all figure panels, statistical calculations were performed using a two-tailed Student's *t*-test (* 0.001 < *P* < 0.05; **P < 0.001). All cells were harvested at ≤ 80% confluency.

In order to determine if elevated *NNMT* expression is a general phenomenon associated with glucose deprivation, we next analyzed glucose-restricted cells generated from two additional epithelial ovarian cancer cells lines, OVCAR4 and OAW28, both of which are considered faithful representatives of HGSC [39]. Similarly to OVCAR3 Gluc cells, OVCAR4 and OAW28 Gluc sublines were unperturbed in low glucose conditions unlike their control cells (Figure 2A). We observed that *NNMT* expression was consistently elevated in all glucose-restricted OAW28 and OVCAR4 cells. In contrast, expression of *SLC2A1* and *G6PD* was increased in glucose-restricted OAW28 cells but not in OVCAR4 cells (Supplementary Figure 1A), suggesting that adaptation of OVCAR4 cells to glucose withdrawal did not depend on the upregulation of those targets. This could be explained by the higher baseline expression level of those targets in parental OVCAR4 cells, which in turn could mediate greater intrinsic adaptability of this cell line to glucose deprivation. Indeed, the comparison of parental OVCAR3, OAW28 and OVCAR4 cells in low density seeding conditions revealed that OVCAR4 cells had the highest level of adaptation to glucose deprivation. Following low density seeding in low glucose, the relative viability of OVCAR4 cells after 18d was the highest among the three cell lines and significantly higher than relative viability of OVCAR3 or OAW28 cells (Figure 2B). This correlated with higher RNA expression of *NNMT* and *G6PD* in OVCAR4 cells compared to the two other cell lines (Figure 2C). Contrary, *SLC2A1* expression was highest in OAW28 cells (Figure 2C) and correlated with the most efficient utilization of sugars such as dextrin, D-fructose and maltotriose in this cell line (Figure 2D). However, utilization of methylated substrates, such as α-methyl-D-galactoside, α-methyl-D-glucoside and mono-methyl succinate, was only observed in OVCAR4 cells with high endogenous expression of *NNMT*. Thus, comparison of endogenous *NNMT* expression with the level of adaptation to glucose deprivation in parental cell lines supports the hypothesis that *NNMT* increases metabolic plasticity of ovarian cancer cells by allowing them to efficiently utilize alternative energy substrates, such as methylated carbohydrates. We next proceeded to test this hypothesis directly by depleting *NNMT* in glucose-restricted cells.

### *NNMT* knockdown reverses glucose independence

**Figure 3:**
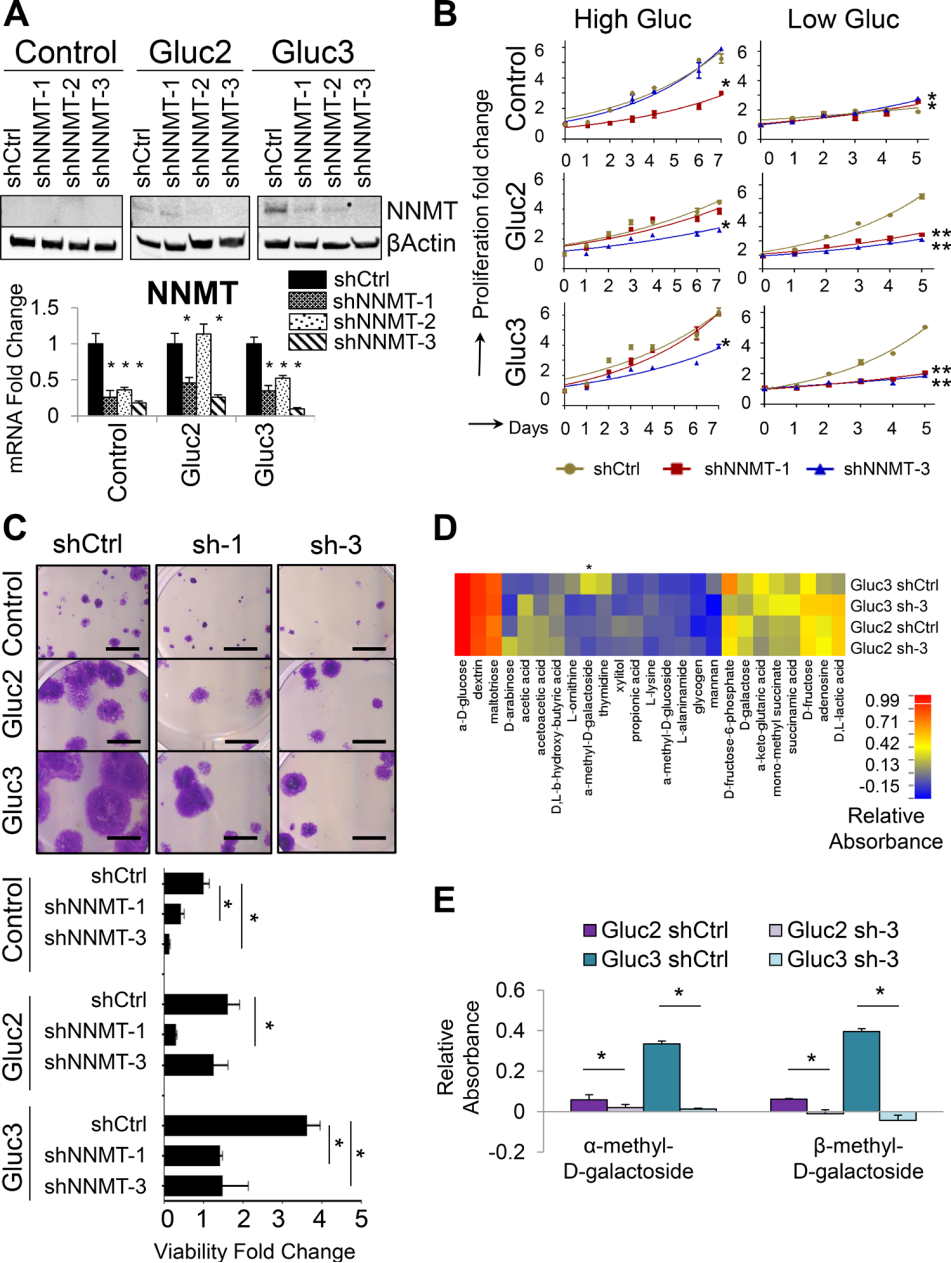
*NNMT* depletion in glucose-restricted OVCAR3 cells reverses tolerance to low glucose levels (**A**) Western blot and qRT-PCR analysis of shRNA-mediated *NNMT* knockdown show decreased NNMT protein and mRNA levels in cells transduced with shNNMT. shNNMT-1 and 3 were the most efficient across all sublines and thus all functional analyses were performed with those two shRNAs. (**B**) Proliferation assays performed over 5–7 d demonstrate that shRNA-mediated *NNMT* knockdown significantly decreased the proliferation capacity of glucose-restricted sublines (Gluc-2 and -3) in low glucose medium compared to shCtrl, whereas the effect on proliferation in the presence of glucose was less prominent. Total number of viable cells was measured by a luminometric viability assay. (**C**) *NNMT* knockdown significantly reduces viability of glucose-restricted sublines and control OVCAR3 cells cultured in medium with low glucose levels. Cells were seeded at low density and allowed to expand for 5 weeks. Scale bar: 5 mm. (**D**) Biolog Microarray results show that *NNMT* depletion partially reverses the metabolic adaptations of glucose-restricted OVCAR3 sublines Gluc-2 and Gluc-3 by decreasing their ability to utilize sugars such as xylitol, mannan, D-galactose, as well as methylated carbohydrate α-methyl-D-galactoside in the absence of glucose. The heatmap illustrates relative substrate utilization, which is defined by an absorbance value normalized by deducting the negative control absorbance (uncoated well) from the absorbance value of a well coated with a given substrate, and dividing it by the average of positive control (wells coated with α-D-glucose). Substrates differentially utilized and showing statistical significance in both Gluc-2 and Guc-3 are marked with asterisks. (**E**) Biolog Microarray results show that *NNMT* knockdown (sh-3) reverses increased efficiency of OVCAR3 Gluc-2 and Gluc-3 sublines to utilize α-methyl-D-galactoside and β-methyl-D-galactoside in the absence of glucose. Relative absorbance was calculated as described above in (D). For all figure panels, statistical calculations were performed using a two-tailed Student's *t*-test (* 0.001 < *P* <0.05; **P < 0.001).

To test if *NNMT* is required for the metabolic adaptations in glucose-restricted cells, we used shRNA delivered via lentivirus to deplete *NNMT* in the glucose-restricted sublines OVCAR3 Gluc-2 and Gluc-3 (Figure 3A). We tested three individual shRNAs targeting *NNMT* and chose two (shNNMT-1 and shNNMT-3) and a non-targeting control shRNA (shCtrl) for further experiments. Depletion of *NNMT* reversed proliferative capacity of OVCAR3 Gluc-2 and Gluc-3 sublines but had no effect on the proliferation of control OVCAR3 cells over the 5-day long culture in low glucose DMEM at normal seeding density conditions (Figure 3B). *NNMT* depletion also reduced viability of glucose-restricted cells seeded at low-density in low glucose conditions (Figure 3C). Interestingly, viability of control OVCAR3 shNNMT cells was also reduced compared to control OVCAR3 shCtrl cells (Figure 3C) after long term culture (5 weeks) in low glucose conditions, suggesting that suppressing *NNMT* induction during prolonged glucose deprivation prevents adaptation to low glucose conditions. In order to assess if the reversal of glucose independence is accompanied by changes in the utilization of alternative substrates, we subjected *NNMT*-depleted OVCAR3 Gluc-2 and Gluc-3 cells to Biolog Phenotype Microarray analysis (Figure 3D). Here we found that utilization of methylated carbohydrates, such as α-methyl-D-galactoside and its isoform, β-methyl-D-galactoside, was significantly decreased in shNNMT cells compared to shCtrl (Figure 3E). Together with our observation that these substrates are only metabolized in *NNMT*-expressing OVCAR4 cells and glucose-restricted OVCAR3 cells, but not control OVCAR3, these results suggest that NNMT is required for the utilization of α-methyl-D-galactoside and other methylated substrates, thus establishing a new role for NNMT in metabolic plasticity required for survival of glucose starvation.

This conclusion was further supported by results from *NNMT*-null cells generated by CRISPR/Cas9 (Supplementary Figure 2A). While CRISPR/Cas9-mediated knockout of *NNMT* did not yield viable colonies in OVCAR3 or its sublines (data not shown), we were able to generate *NNMT*-null OVCAR4 cells (Supplementary Figure 2A). Loss of *NNMT* expression completely abolished OVCAR4 growth in low glucose medium upon culturing in low seeding density conditions for 14 days. Similarly, we used CRISPR/Cas9 to deplete *NNMT* in SKOV3 ovarian cancer cells (endometrioid/clear cell subtype [40]), a cell line previously shown to have high *NNMT* activity [41]. As expected, *NNMT* depletion in SKOV3 cells resulted in greatly diminished ability of this cell line to grow in low glucose medium (Supplementary Figure 2A). In summary, these results suggest that NNMT is required for glucose independence in ovarian cancer cell lines and supports cell survival in glucose-deprived environment by facilitating metabolic adaptations.

### ZEB1 is an upstream regulator of *NNMT* and mediator of glucose independence

**Figure 4:**
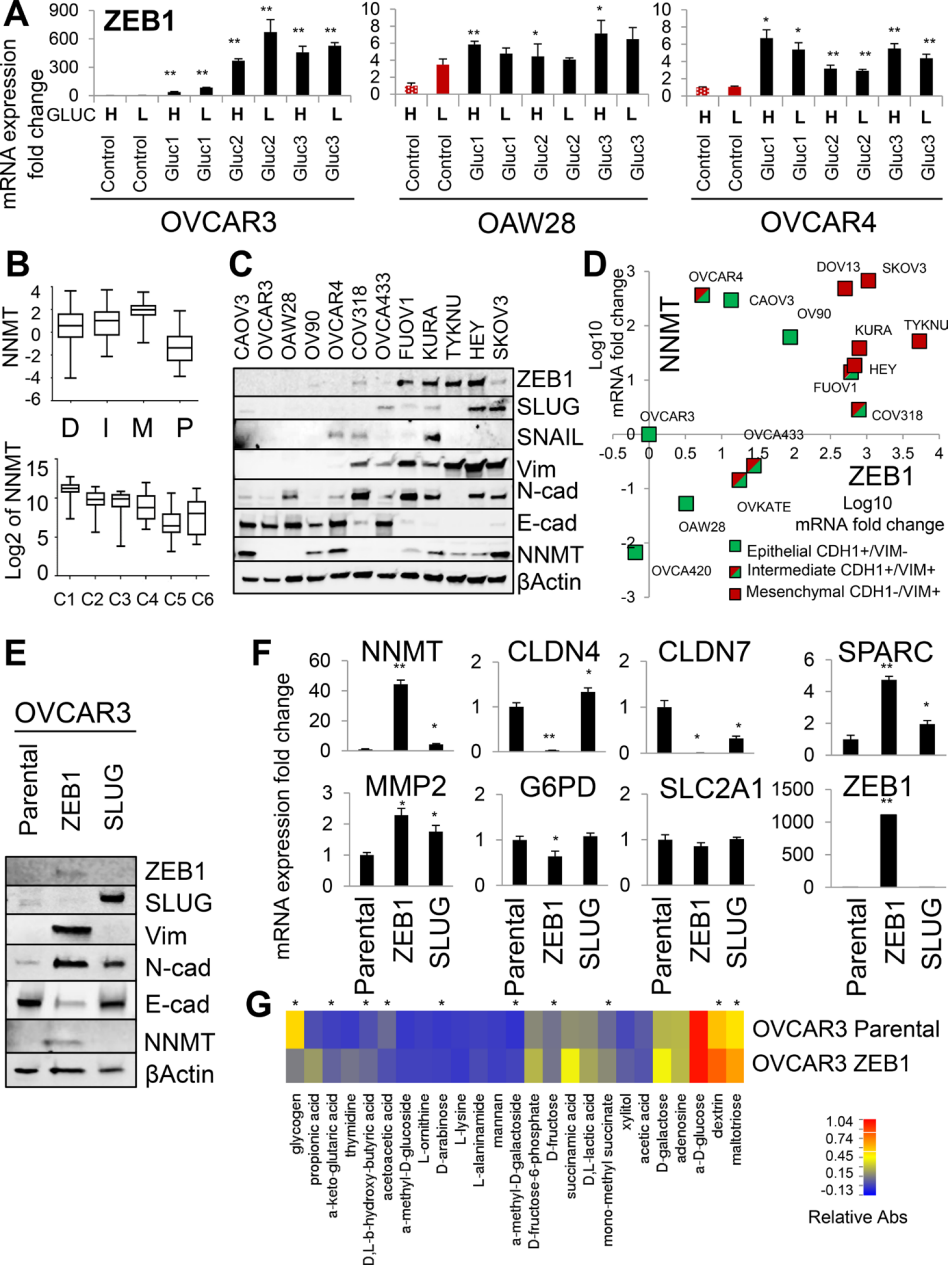
ZEB1 is an upstream regulator of NNMT and mediator of glucose independence (**A**) qRT-PCR shows that mRNA expression of *ZEB1* is consistently upregulated in all glucose-restricted sublines generated from OVCAR3, OVCAR4 and OAW28 cell lines. (**B**) *NNMT* expression correlates with the mesenchymal subtype of HGSC patients (M) as defined by the Cancer Genome Atlas [32] and the C1 subtype of HGSC patients, characterized by the reactive stroma gene signature [33]. (**C**) NNMT protein expression is consistently higher in mesenchymal-like ovarian cancer cell lines (low E-cadherin; high vimentin, ZEB1 or SLUG expression: KURAMOCHI, TYKNU, HEY, SKOV3, FUOV1) compared to epithelial-like cell lines (high E-cadherin; low vimentin, ZEB1 or SLUG expression: OVCAR3, OVCA433, OAW28, COV318). However, some epithelial-like cell lines, such as CAOV3, OV90 and OVCAR4, demonstrate relatively high NNMT levels. (**D**) *NNMT* mRNA expression positively correlates with elevated *ZEB1* and *Vim* (vimentin) expression, and shows a tendency to negatively correlate with *CDH1* (E-cadherin) expression in ovarian cancer cell lines. (**E**) Western blot analysis shows that parental OVCAR3 cells ectopically expressing ZEB1 undergo epithelial-to-mesenchymal transition (EMT), as manifested by decreased expression of E-cadherin and increased expression of N-cadherin and vimentin. Overexpression of ZEB1, but not SLUG, induced NNMT protein expression. (**F**) qRT-PCR analysis shows that ZEB1 overexpression in OVCAR3 cells induces *NNMT, MMP2, SPARC* and decreases *CLDN4* and *CLDN7* expression, but has no effect on the *SLC2A1* and *G6PD* expression. (**G**) Biolog Microarray studies shows that ectopic *ZEB1* expression recapitulates metabolic adaptations observed in OVCAR3 glucose-restricted cells, such as increased utilization of sugars (D-galactose, dextrin, maltotriose, xylitol, D-fructose), ketones (D, L-β-hydroxy-butyric acid), D, L-lactic acid and methylated substrates (α-methyl-D-galactoside, α-methyl-D-glucoside, mono-methyl succinate) in the absence of glucose. Asterisks (*) denote substrates differentially utilized between control and transformed cells demonstrating statistical significance *P* < 0.05. For all figure panels, statistical calculations were performed using a two-tailed Student's *t*-test (* 0.001 < *P* < 0.05; ** *P* < 0.001).

Since *NNMT* expression is absent in both OVCAR3 and OAW28 epithelial ovarian cancer cell lines but highly induced in glucose-restricted sublines, we next sought to determine the upstream mechanisms controlling NNMT expression. RNAseq analysis in OVCAR3 (Supplementary Table 1) and subsequent qRT-PCR validation of individual candidate genes in all glucose-restricted cell lines and controls revealed consistent upregulation of *ZEB1, MMP2, SPARC* and *CTGF* in glucose-restricted sublines (Figure 4A and Supplementary Figure 3A). These genes are associated with epithelial-to-mesenchymal transition (EMT) and indeed, we found the highest *NNMT* expression in the mesenchymal subtype of HGSC [32, 33] (Figure 4B). In our panel of ovarian cancer cell lines, NNMT protein expression positively correlated with expression of ZEB1 and other mesenchymal markers, such as vimentin (Figure 4C). Also, all ovarian cancer cell lines with high ZEB1 expression were positive for NNMT (Figure 4C and 4D). However, some cell lines with epithelial morphology (positive for E-cadherin, negative for vimentin and ZEB1, e.g. CAOV3 and OV90) were still positive for NNMT (Figure 4C and 4D), suggesting that ZEB1-independent mechanism can also induce NNMT. To test directly if ZEB1 mediates some of the transcriptional changes observed in glucose-restricted cells, we overexpressed ZEB1 and SLUG in OVCAR3 cells and compared them to parental control cells (Figure 4E). Only ZEB1 robustly induced *NNMT* expression and transcriptional changes reminiscent of EMT, such as loss of E-cadherin and claudins (*CLDN4*, *CLDN7*), and induction of mesenchymal markers, such as N-cadherin, vimentin, *SPARC* and *MMP2* (Figure 4E and 4F). Interestingly, ectopic ZEB1 expression had no effect on the expression of *G6PD* and *SLC2A1* (Figure 4F), suggesting that upregulation of those targets in glucose-restricted cells is ZEB1-independent. Ectopic ZEB1 expression in OVCAR4 cells induced similar transcriptional changes (Supplementary Figure 3B and 3C). However, relative upregulation of *NNMT* was less prominent in OVCAR4-ZEB1 cells, consistent with higher baseline expression of NNMT in OVCAR4 compared to OVCAR3 cells.

Next, we tested if ZEB1 overexpression can recapitulate the metabolic adaptations seen in glucose-restricted cells. To this end, we compared parental OVCAR3 cells and OVCAR3-ZEB1 cells using Biolog Phenotype Microarrays (Figure 4G). Indeed, OVCAR3-ZEB1 cells were able to more efficiently utilize methylated substrates, such as α-methyl-D-galactoside and mono-methyl succinate, as well as some additional carbohydrates previously correlated with glucose-independence, such as maltotriose (Figure 4G). We validated these results by overexpressing ZEB1 in another ovarian cancer cell line of serous histology (OVCA433), which shows no detectable endogenous expression of NNMT or ZEB1 (Supplementary Figure 4B [45]). OVCA433-ZEB1 cells express NNMT and other mesenchymal markers (Supplementary Figure 4B) and proliferate at higher rates in low glucose conditions relative to OVCA433-GFP control cells (Supplementary Figure 4C). Biolog analysis revealed that utilization of methylated substrates was also increased in OVCA433-ZEB1 cells (Supplementary Figure 4D). We conclude that ectopic *ZEB1* expression is sufficient to upregulate NNMT and other mesenchymal genes, enable cells to use methylated substrates as an alternative energy sources, and induce glucose independence in glucose-dependent epithelial ovarian cancer cell lines.

### Glucose deprivation creates phenotypic heterogeneity

**Figure 5:**
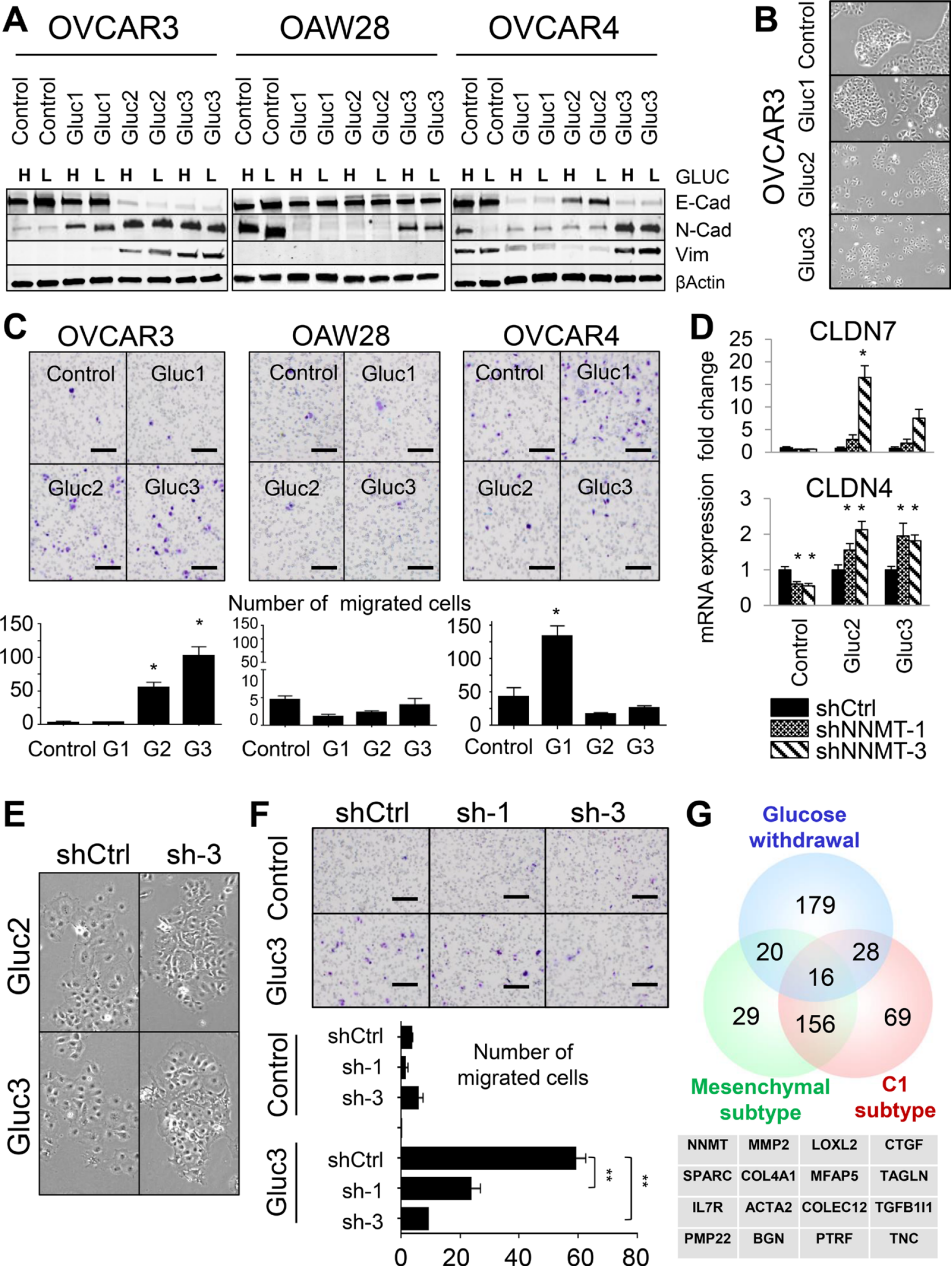
Glucose deprivation creates phenotypic heterogeneity in cellular populations (**A**) EMT is not a prerequisite for glucose independence. While some glucose-restricted cells acquire a mesenchymal-like phenotype, as manifested by the loss of E-cadherin and gain of Vimentin and N-cadherin expression (OVCAR3 Gluc-2 and Gluc-3), other sublines assume epithelial/mesenchymal hybrid state (OVCAR3 Gluc-1, OVCAR4 Gluc-1 and Gluc-3) or retain their epithelial morphology (OVCAR4 Gluc-2, all OAW28 Gluc sublines). (**B**) OVCAR3 glucose-restricted Gluc-2 and Gluc-3 loose cell-cell interactions compared to control and Gluc-1 cells. (**C**) OVCAR3 Gluc-2 and Gluc-3 sublines acquire migratory properties compared to control cells, as assessed by standard transwell migration assays. Only OVCAR4 Gluc-1 cells acquire more migratory potential compared to OVCAR4 control cells. OAW28 glucose-restricted sublines did not gain migratory capabilities. Scale bar: 100 μm. (**D**) *NNMT* knockdown in OVCAR3 Gluc-2 and Gluc-3 cells increases *CLDN4* and *CLDN7* expression. (**E**) *NNMT* depletion in OVCAR3 Gluc-2 and Gluc-3 cells partially restores their original morphology and ability to form compact colonies. (**F**) Genetic depletion of *NNMT* significantly reduces the migratory potential of OVCAR3 Gluc-3 cells. Scale bar: 100 μm. (**G**) Genes upregulated upon chronic glucose withdrawal partially overlap with the mesenchymal and C1 subtypes of HGSC. Table shows 16 genes commonly upregulated in those signatures, including *NNMT* and classic mesenchymal genes such as *MMP2, LOXL2, CTGF, SPARC, ACTA2* and *TAGLN*. For all figure panels, statistical calculations were performed using a two-tailed Student's *t*-test (* 0.001 < *P* < 0.05; ** *P* < 0.001).

ZEB1 is a potent inducer of EMT [46–48] and overexpression of ZEB1 induced EMT in epithelial OVCAR3 cells, as determined by loss of E-cadherin protein expression and induction of mesenchymal markers, such as vimentin and N-cadherin (Figure 4E). OVCAR3-ZEB1 cells also had increased migratory potential, as demonstrated by Boyden chamber migration assays (Supplementary Figure 4A). Therefore, we considered the possibility that EMT may be a consequence of glucose deprivation. Indeed, loss of E-cadherin and induction of N-cadherin and vimentin was observed in OVCAR3 Gluc-2 and Gluc-3 sublines (Figure 5A). In addition, both sublines lost expression of genes involved in maintaining cell-cell junctions, such as *PKP3, CLDN4, CLDN7* (Supplementary Figure 5A), *EPCAM, CDH1* and *OCLN* (Supplementary Table 1). As a result, OVCAR3 Gluc-2 and Gluc-3 cells lost cell-cell interactions and grew as loosely connected cell populations, in contrast to control OVCAR3 that formed tightly linked patches of cells (Figure 5B). Gene Ontology (GO) analysis of RNAseq results for these cell lines also revealed activation of several processes associated with EMT [49, 50], such as cell migration, anti-apoptotic signaling and vasculature development (Supplementary Table 3). In contrast, downregulated cellular functions included positive regulation of mitosis, epithelium development and cell-cell adhesion. Furthermore, Gene Set Enrichment Analysis revealed that the transcriptional changes observed in OVCAR3 Gluc-2 and Gluc-3 cells were enriched for both an EMT signature gene set and genes, which expression is downregulated in cancer cells upon co-culture with activated stroma [51] (Supplementary Table 4 and Supplementary Figure 5B). This data supports the hypothesis that OVCAR3 Gluc-2 and Gluc-3 undergo EMT, similar to what has been shown for another form of metabolic stress, hypoxia [52–55].

However, the OVCAR3 Gluc-1 subline, which had an intermediate metabolic phenotype (*i.e*. was less proliferative in low glucose DMEM and had lower NNMT levels), did not undergo full EMT. While OVCAR3 Gluc-1 cells became positive for N-cadherin, they remained negative for vimentin and retained expression of E-cadherin (Figure 5A), as well as *PKP3, CLDN4* and *CLDN7* (Supplementary Figure 5A). Also, OVCAR3 Gluc-1 cells morphologically resembled control OVCAR3 (Figure 5B). Taken together, these data suggested that EMT may be a consequence but not a prerequisite of glucose independence. Analysis of the additional glucose-restricted cell lines confirmed this observation: western blot and qRT-PCR analysis of the glucose-restricted OVCAR4 sublines revealed loss of E-cadherin, claudins (*CLDN4*, *CLDN7*) and plakophilin (*PKP3*), but in addition, loss of N-cadherin and vimentin in two out of three sublines (Figure 5A and Supplementary Figure 5A). In OAW28, we detected virtually no changes in classic EMT-associated gene expression: E-cadherin and vimentin protein levels were similar in glucose-restricted sublines and control cells (Figure 5A), and RNA expression of *CLDN4, CLDN7* and *PKP3* remained unchanged or were elevated in some sublines (Supplementary Figure 5A). We next assessed migratory potential in all glucose-restricted cell lines as a functional property associated with EMT (Figure 5C). Two out of three OVCAR3 sublines (OVCAR3 Gluc-2 and Gluc-3) and one out of three OVCAR4 sublines (OVCAR4 Gluc-1) became more migratory, while the remaining glucose-restricted sublines, including all OAW28 sublines, did not (Figure 5C). Thus, we conclude that EMT is not required for glucose independence, but glucose independence may be associated with transcriptional and functional changes indicative of EMT, depending on the level of ZEB1 induction. Furthermore, glucose independence generates phenotypic heterogeneity, resulting in populations with various degrees of mesenchymal properties.

### NNMT maintains the mesenchymal-like phenotype associated with glucose independence

Since NNMT has been reported as a metabolic mesenchymal signature gene [56] with potential roles in migration [57] and aggressive tumor behavior [58, 59], we next asked if in addition to its requirement for glucose independence, NNMT also plays a role in maintaining mesenchymal-like features in glucose-restricted cells. We found that NNMT depletion elevated expression of *CLDN4* and *CLDN7* (Figure 5D), as well as partially rescued the loss of cell–cell interactions in OVCAR3 Gluc-2 and Gluc-3 cells (Figure 5E). In addition, *NNMT* depletion significantly decreased the migratory potential of OVCAR3 Gluc-2 and Gluc-3 cells compared to shCtrl (Figure 5F), while it had no effect in epithelial OVCAR3 controls.

We then considered the possibility that glucose independence may be associated with a mesenchymal gene signature that is different from the classic EMT signature. To address this possibility, we compared significantly upregulated genes in OVCAR3 Gluc-2 and Gluc-3 cells (Supplementary Table 1) with published signatures of the mesenchymal (or desmoplastic) subtype of HGSC [32, 33] (Figure 5G and Supplementary Table 5). Among the 16 genes common to these signatures, *NNMT, LOXL2, SPARC, MMP2*, *TAGLN, ACTA2* and *CTGF*, are known mesenchymal regulators and were commonly upregulated in all glucose-restricted sublines (Supplementary Figure 3A and Supplementary Figure 5A), suggesting that mesenchymal gene signature associated with glucose independence may be expressed in E-cadherin positive, vimentin-negative cells. Interestingly, the high baseline expression level of some of these genes in parental OVCAR4 cells (Supplementary Figure 5C) correlates with their resistance to glucose deprivation compared to other epithelial ovarian cancer cell lines, such as OVCAR3 and OAW28. Of note, array-CGH (Figure 1F) revealed DNA copy number gains for *LOXL2, TAGLN* and *MMP2* in glucose-restricted OVCAR3 cells, implying that these genomic events may further contribute to the transcriptional changes observed in glucose-restricted cells.

### NNMT is expressed in human HGSC

Finally, we sought to assess NNMT protein levels in human HGSC. Analysis of published datasets revealed that *NNMT* expression is highest in the mesenchymal subtype of ovarian cancer (Figure 4B), which some studies have associated with the worst overall survival [32, 33]. In line with this, our survival analysis of over 922 publicly available HGSC [60] showed that *NNMT* expression is negatively correlated with both overall and progression-free survival (Supplementary Figure 6A and 6B) in HGSC patients (stage III and IV). Also, a broad comparison of *NNMT* expression levels in tumors of various origin (red bars) and corresponding normal control tissues (green bars) showed a general trend toward higher *NNMT* expression in cancer than in normal tissue (Supplementary Figure 6C). Specifically, carcinomas of ovary, cervix, vulva and vagina, all showed significantly upregulated NNMT expression compared to healthy tissues.

**Figure 6:**
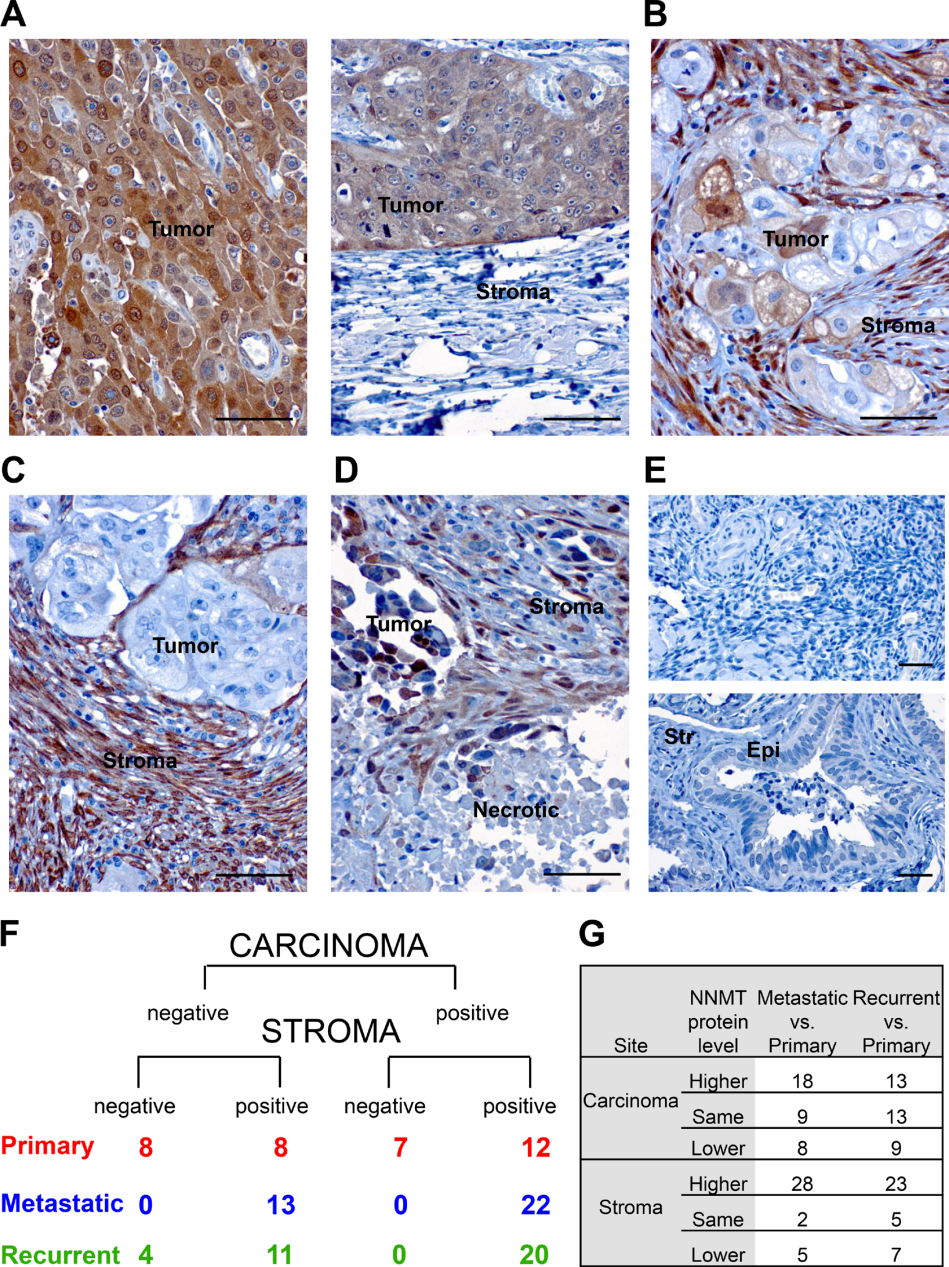
NNMT expression in human HGSC (**A**) Immunohistochemical analysis of NNMT protein expression in ovarian carcinomas shows a heterogeneous expression pattern. In individual HGSC specimens, NNMT protein was detected either in tumor compartment; (**B**) both in tumor and adjacent stroma; (**C**) exclusively in cancer stroma; and (**D**) frequently next to necrotic tumor regions. (**E**) NNMT expression was undetectable in normal ovary (top) and fallopian tube epithelium (Epi) and stroma (Str) (bottom). (**F**) NNMT immunohistochemistry on tissue microarray consisting of patient-matched primary, recurrent and metastatic specimens demonstrates that most of the ovarian cancer specimens, which express NNMT in the tumor compartment, also have detectable NNMT protein in the stroma. However, many specimens show high NNMT levels only in cancer stroma. Also, a significant portion of primary carcinomas demonstrate non-detectable NNMT expression both in stroma and tumor, while all metastatic specimens express NNMT in stroma. (**G**) In most of the analyzed patients NNMT protein expression in stroma is significantly elevated in metastatic and recurrent biopsies compared to patient-matched primary carcinomas. However, NNMT levels in the carcinoma compartment are elevated only in metastases compared to matched primary specimens, but not in recurrent biopsies. Scale bar: 50 μm.

Taken together, these *in silico* results prompted us to assess NNMT protein expression in primary, metastatic and recurrent ovarian carcinomas. For this purpose, we performed immunohistochemistry (IHC) for NNMT on a tissue microarray (TMA) containing 35 triplets of matched primary, recurrent and metastatic HGSC. Our results show that NNMT protein expression is detectable both in the carcinoma and adjacent stroma (Figure 6A–6D), with NNMT expression in some specimens exclusively present only in one of the compartments (Figure 6A and 6C). Furthermore, elevated NNMT was often detected in close proximity to the necrotic tissue sites (Figure 6D). Of note, normal ovary and fallopian tube epithelium or stroma had no detectable NNMT levels (Figure 6E). Also, NNMT was expressed in 54% of primary (*n* = 19 positive versus *n* = 16 negative), 63% metastatic (*n* = 22 positive versus *n* = 13 negative) and 57% recurrent carcinomas (*n* = 20 positive versus *n* = 15 negative) (Figure 6F). Detailed analyses of intratumoral differences in NNMT expression between primary, recurrent and metastatic specimens demonstrate that NNMT expression was specifically elevated in metastatic biopsies compared to corresponding primary carcinoma sites (*n* = 18 expressed NNMT higher than matched primary, *n* = 9 had the same expression, *n* = 8 had lower expression) (Figure 6G). In recurrent biopsies, most of the patients showed higher (*n* = 13) or the same (*n* = 13) NNMT protein level, whereas only 9 patients had decreased NNMT expression compared to matched primary carcinoma. Interestingly, NNMT levels in the stroma compartment were consistently upregulated both in the metastatic (*n* = 28 expressed NNMT higher than matched primary, *n* = 2 had the same expression, *n* = 5 had lower expression) and recurrent biopsies (*n* = 23 expressed NNMT higher than matched primary, *n* = 5 had the same expression, *n* = 7 had lower expression) compared to primary carcinomas. In summary, we found that more than half of all primary HGSC contained NNMT-positive regions. Moreover, NNMT was further induced in metastatic and recurrent HGSC compared to their matched primary tumors.

## DISCUSSION

### Metabolic adaptations in glucose-restricted ovarian cancer cells

High-grade serous ovarian cancer (HGSC) ranks among the five deadliest cancers in women and is the second most common gynecological cancer. It remains a burden for women worldwide and its low 5-year survival rate (∼40%) has not improved significantly in the past 40 years. This is partly due to complex molecular and phenotypic disease heterogeneity and inherent drug resistance. Specifically, the mesenchymal subtype of HGSC correlates with the poorest overall survival [33, 61] and extensive desmoplastic stromal reaction. Furthermore, the transition to mesenchymal-like subtype was observed in chemoresistant HGSC [62]. We hypothesized that metabolic alterations in the tumor microenvironment, such as glucose starvation, may have direct phenotypic consequences on ovarian cancer evolution. Glucose deprivation is frequently observed in advanced human malignancies, such as HGSC [15], as a result of intrinsic intratumoral heterogeneity in the vasculature development or following anti-angiogenic treatment [18–21]. Such nutritional stress may select for highly metabolically plastic cells and alter the molecular subtype of cancer cells surviving the initial shock of glucose deprivation. This prompted us to investigate mechanisms and consequences of adaptation to chronic low glucose levels in epithelial ovarian cancer cell lines. We identified the methyl transferase NNMT as a novel regulator of metabolic plasticity in cells adapted to low glucose levels. Our results suggest that NNMT plays a crucial role in adaptation to glucose deprivation, in addition to the glucose transporter, SLC2A1 (GLUT1), and the enzyme glucose-6-phosphate dehydrogenase, G6PD, which were previously described in the context of glucose independence [25, 63] and confirmed in our studies.

In line with increased *SLC2A1* expression, we observed that glucose-restricted cells can utilize other sugars, such as D-fructose, D-arabinose, mannan, maltotriose and dextrin more efficiently than control cells. We also observed increased use of ketone bodies (acetoacetic acid and D, L-β-hydroxy-butyric acid) in glucose-restricted cells. Ketone bodies together with lactic acid can fuel the Tricarboxylic Acid Cycle (TCA cycle) and produce NADH and FADH_2_. These molecules feed oxidative phosphorylation (OXPHOS) to produce ATP. Thus, ketosis may represent an alternative way to produce energy by glucose-restricted sublines. However, as a consequence of elevated OXPHOS activity, levels of reactive oxygen species (ROS) rise [64], which need to be offset by PPP activity. The main function of PPP is the production of reducing agents and pentose phosphates for nucleic acid and lipid synthesis [28–30], and G6PD is a rate-limiting enzyme of the PPP, which generates NADPH required to reduce oxidized glutathione (GSSG) to reduced glutathione (GSH). GSH is required for the reduction of ROS and the maintenance of the normal redox state [64]. Thus, elevated expression of G6PD may help glucose-deprived cells survive oxidative stress and rewire the metabolism. Of note, elevated expression of *SLC2A1* and *G6PD*, as well as increased PPP and OXPHOS activity, have recently been linked with resistance of putative ovarian cancer stem cells to glucose deprivation [25]. Consistent with this study, we observed that glucose-restricted cells exhibit increased expression of ovarian cancer stem cell markers, such as *CD117 (KIT* [35] and *CD44* [35], as well as decreased expression of *CA125 (MUC16)* [38].

Both *G6PD* and *NNMT* are subject to DNA copy number gains and transcriptional upregulation in glucose-restricted cells, suggesting that they play non-overlapping functions in glucose independence. NNMT methylates nicotinamide using S-adenosylmethionine (SAM) as a methyl donor, thereby creating the stable metabolite, S-adenosylhomocysteine (SAH) and reducing the amount of methyl groups available for cellular methylation reactions [41, 42]. As a result, NNMT plays a fundamental role in regulating cellular methylation potential and its elevated expression may induce epigenetic remodeling of cells by altering histone methylation status [41, 65]. We found that NNMT was required for the utilization of methylated substrates, such as α-methyl-D-galactoside, α-methyl-D-glucoside and mono-methyl succinate, as alternative sources of energy in glucose-restricted cells. These substrates were poorly utilized in NNMT-low expressing epithelial ovarian cancer cells, suggesting that NNMT-deprived cells may not be able to efficiently metabolize these methylated compounds in the absence of glucose. Considering the role of NNMT in methylation reactions, it is plausible that some of those substrates could serve as potential methyl donors for NNMT-mediated enzymatic reactions. In line with this, *NNMT* depletion significantly decreased the ability of glucose-restricted sublines to utilize α-methyl-D-galactoside and its isoform, β-methyl-D-galactoside. However, little is known about the biochemistry of methylated carbohydrates and further studies are needed to determine if and how these compounds are able to fuel metabolic pathways.

Recent studies also classified *NNMT* as a Mesenchymal Metabolic Signature Gene, commonly expressed at a high level in the human mesenchymal-like cancer cells (*n* = 978) [56]. Our data also show that NNMT expression is highest in the mesenchymal subtype of ovarian cancer. Previous studies also showed that NNMT expression is elevated in “aggressive” cancer cell lines [41] and NNMT has a role in regulation of cellular migration [41, 57, 66]. In line with these results, *NNMT* knockdown decreased proliferative and migratory properties of glucose-restricted OVCAR3 sublines. In addition, *NNMT* depletion in parental ovarian cancer cell lines with high baseline *NNMT* expression, such as SKOV3 and OVCAR4, caused a drastic decrease in their viability in low glucose conditions.

### ZEB1-mediated induction of NNMT links metabolic stress to mesenchymal gene expression

We found that chronic glucose deprivation in several epithelial ovarian cancer cell lines induces mesenchymal gene expression. Although we found that some glucose-restricted cells lose cell-cell interactions and become more migratory, the extent of mesenchymal transformation in response to glucose deprivation was variable and most glucose-restricted ovarian cancer sublines retained their epithelial morphology. In fact, we observed considerable phenotypic heterogeneity in the epithelial/mesenchymal status of glucose-restricted sublines generated from the same cell line. Therefore, we conclude that full EMT is not a prerequisite for survival of prolonged glucose deprivation. Instead, we identified a set of mesenchymal genes such as *MMP2*, *SPARC*, *CTGF* and *NNMT*, that were consistently upregulated in glucose-restricted cells and shared with the mesenchymal subtype of HGSC described by Tothill *et al*. [33] and TCGA [32]. This set of mesenchymal genes was associated with glucose independence even when classic epithelial markers, such as E-cadherin, were retained. Furthermore, *ZEB1* was consistently upregulated in all glucose-restricted sublines and its ectopic expression partially recapitulated glucose independence and mesenchymal-like gene expression observed in Gluc cells, in particular elevated *MMP2, SPARC*, and *NNMT* expression. However, ZEB1 did not upregulate expression of these genes to the level observed in glucose-restricted cells (2-40-fold change by ZEB1 overexpression in OVCAR3 compared to 20-200-fold change in glucose-restricted OVCAR3 sublines), suggesting that additional transcriptional regulators contribute to mesenchymal-like gene expression in glucose-restricted cells. Furthermore, ZEB1 did not alter expression of *G6PD* and *SLC2A1*, which supports the notion that these metabolic genes are regulated by independent mechanisms. Thus, whether or not EMT occurs in glucose-restricted cells appears to be dependent on the level of *ZEB1* induction, as well as the activation status of alternative pathways contributing to glucose independence. For example, high endogenous expression of the glucose transporter *SLC2A1* in OAW28 cells may reduce the requirement for *ZEB1* and *NNMT*, and indeed, the level of upregulation of those targets was moderate in glucose-restricted OAW28 cells compared to their drastic induction in glucose-restricted OVCAR3 cells.

Genomic events also seem to contribute to mesenchymal-like gene expression changes in glucose-restricted cells. Some of the most significantly upregulated genes, such as *NNMT, MMP2, LOXL2*, and *TAGLN*, gained one or more DNA copies in the process of acquired glucose independence. Thus, nutritional stress may select for transcriptional and genomic events in ovarian cancer cells to elicit mesenchymal-like gene expression changes. Of note, glucose-restricted sublines have comparable passage number to control cells, which were simultaneously passaged throughout the eight-month-period and show the same proliferation rate in glucose-containing medium as glucose-restricted cells in low glucose medium (Figure 1B). Thus, the difference in the number of population doublings could not account for the observed genomic instability or metabolic alterations.

In addition, our data show that NNMT may play a crucial role in ovarian cancer progression or metastases since its expression both in tumor and stroma compartment is significantly increased in metastatic and recurrent carcinomas compared to patient-matched primary tumor specimens. Consistently with our results, studies by Bignotti *et al*. [67] and Brodsky *et al*. [68] identified *NNMT* as a part of a signature of significantly upregulated genes in omental metastasis compared to primary HGSC.

In summary, our results suggest that the ZEB1/NNMT signaling axis induces phenotypic and metabolic plasticity, as well as mesenchymal gene expression in ovarian cancer cells upon chronic glucose deprivation. Since heterogeneity of tumor vasculature is a common feature of advanced malignancies, it is plausible that individual ovarian cancers, irrespective of their expression subtype, may acquire regions of highly metabolically plastic, mesenchymal-like cancer cells due to exposure to nutritional stress. These cells may then contribute to metastasis and recurrence. Of note, one of the mechanisms of acquired bevacizumab resistance in glioblastoma [16] and in colorectal cancer cells [17] is EMT. While our studies suggest that EMT is not a requirement for glucose independence, it may represent a phenotypic outcome of adaptation to nutritional stress as a direct consequence of ZEB1 activity. Furthermore, results of our studies suggest that NNMT is required for the resistance to glucose deprivation and thus, bevacizumab treatment may select for NNMT overexpressing cells. In fact, recent studies showed that *NNMT* expression is significantly elevated in tumors treated with bevacizumab and remains elevated even upon removal of anti-angiogenic therapy [16]. Since bevacizumab is now FDA-approved for HGSC, it will be interesting to see, if NNMT indeed plays a role in drug resistance in this context.

## MATERIALS AND METHODS

### Cell culture and reagents

Human ovarian carcinoma cell lines CAOV3, OVCAR3, OV90 and SKOV3 were obtained from ATCC (Manassas, VA). OVCAR4 cells were purchased from NCI (Frederick, MD). OAW28, HEY and DOV13 were kindly provided by Dr. D. J. Slamon (Los Angeles, CA). OVCA420 and OVCA433 were provided by Dr. R. Drapkin (Boston, MA). Glucose-restricted cells were generated from three ovarian cancer cell lines (OVCAR3, OVCAR4 and OAW28) of serous histology [39] by passaging cells for over eight months in low glucose DMEM medium (Gibco, Thermo Fisher Scientific, Waltham, MA) supplemented with 110 mg/l sodium pyruvate, 10% FBS, 1% antibiotic-antimycotic (Life Technologies, Carlsbad, CA) and 2.5 μg/ml plasmocin (Invivogen). Since FBS contains glucose in the concentration of about 1.25 g/l, the final glucose level in media used by glucose-restricted cells was around 0.125 g/l (0.69 mM). Control cells were maintained for eight months in DMEM medium with high glucose levels (4.5 g/l ≈ 25 mM) (Corning, Corning, NY) supplemented with 10% FBS, 1% antibiotic-antimycotic (Life Technologies) and 2.5 μg/ml plasmocin (Invivogen, San Diego, CA). Cells were cultured at 37°C and 5% CO_2_ in a humidified atmosphere. STR profiling was used to authenticate each cell line (Laragen, Culver City, CA).

### Lentiviral constructs

shRNA constructs targeting *NNMT* and control shRNA were purchased from Sigma-Aldrich (St. Louis, MO) as a glycerol bacterial stock. Bacteria were grown overnight in LB medium supplemented with 100 μg/ml ampicillin (Sigma-Aldrich) at 37°C. shRNA constructs were extracted using Plasmid Maxi Kit (Qiagen, Valencia, CA). Single guides (sg) targeting the first coding exon of *NNMT* were designed using The CRISPR Design web tool [69]. Transfection constructs were prepared as previously described [70, 71]. sgNNMT were ligated into CRISPR/Cas9 lenti V2 plasmids containing the puromycin resistance gene. Transfection of 293T human embryonic kidney cells with shNNMT or sgNNMT (CRISPR/Cas9) and lentiviral packaging plasmids was performed using Lipofectamine 2000 (Invitrogen, Carlsbad, CA) in OptiMEM (Life Technologies). Viral supernatant from each transfection was filtered through 0.45 μm filter unit, supplemented with 5 μg/ml polybrene (Gibco) and added to target cells (OVCAR3 control and Gluc sublines, OVCAR4 and SKOV3 cell lines). Transduced cells were maintained in their appropriate low or high glucose medium and selected with 5μg/ml puromycin (Gibco).

### Western blot analysis

Western blot and protein detection was performed as previously described [72]. The following primary antibodies were used: NNMT (Santa Cruz, Dallas, TX); Vimentin (Thermo Fisher Scientific); E-cadherin and N-cadherin (BD Biosciences, San Jose, CA); ZEB1, SNAIL and SLUG (Cell Signaling, Danvers, MA), and β-Actin (Sigma-Aldrich).

### Quantitative PCR (qPCR)

Total RNA and genomic DNA isolation, cDNA generation and qPCR were performed as previously described [72]. Sequences of primers used for quantitative Reverse Transcriptase and genomic PCR (qRT-PCR and genomic qPCR) are listed in Supplementary Table 6. qRT-PCR results are represented as fold change mRNA expression (2^-ΔΔct) and statistically significant differences between different samples are marked with asterisks (* for 0.001 < *P* < 0.05 and ** for *P* < 0.001). Statistics were calculated using a two-tailed t test.

### Viability assays

To create growth curves, cells were seeded in 96-well plates (1 × 10^3^ per well) in DMEM medium with and without glucose. Cells were harvested daily for six consecutive days using a luminometric assay performed according to the manufacturer's protocol (CellTiter-Glo Luminescent Cell Viability Assay, Promega, Madison, WI). Luminescence was measured after 15 min incubation with the luminescent substrate and scanned using Glomax Multi Detection System (Promega). Relative viability was calculated as described before [73]. Graphs were generated using GraphPad Prism version 6 software (San Diego, CA). Cell viability was also assessed by crystal violet staining. For low seeding density conditions, cells were seeded at the density 3 × 10^3^ cells per 35 mm well of a six-well plate and cultured for 2–5 wk in DMEM medium with and without glucose. Cells were later washed with PBS, fixed in 4% formaldehyde and stained with 0.1% crystal violet. Quantitation was performed by extracting the crystal violet dye with 10% acetic acid and measuring the absorbance at 550 nm with the Glomax Multi Detection System (Promega). At least two independent experiments were performed in triplicate for each cell line for all proliferation and viability assays. All cells were harvested at ≤ 80% confluency.

### Boyden chamber migration assay

Cells were trypsinized with 0.05% Trypsin-EDTA solution without glucose (Gibco), washed twice with PBS and resuspended in DMEM without FBS supplemented with 1% antibiotic-antimycotic and 2.5 μg/ml plasmocin. 5 × 10^4^ cells were seeded in 100 μl DMEM without FBS inside the 24-well Millicell Hanging Cell Culture PET Insert of 8μm pore diameter (Millipore, Billerica, MA) and allowed to settle for 30 minutes at 37°C. 650 μl DMEM supplemented with 10% FBS (attractant) was then added carefully to the well, outside of the insert. After a 24 h-long incubation, cells were washed with PBS, fixed and stained with Diff-Quick Stain Set (Siemens Healthcare Diagnostics, Deerfield, IL). The inside of the insert was cleaned with Q-tip cotton swabs to remove cells, which did not migrate. Membranes were then cut out of the inserts, mounted on glass slides and imaged on bright field using an upright light microscope.

### Immunofluorescence

Cells were seeded at 1 × 10^5^ density on the sterile round glass cover slips (20 mm diameter) in a 12-well cell culture plate. After 48 h cells were washed with PBS, fixed with 4% paraformaldehyde at room temperature (RT) for 20 min and permeabilized with 0.1% Triton-X-100 solution in PBS for 4 min. After three subsequent PBS washes, samples were blocked with 1% BSA solution in PBS for 1 h. Cells were then incubated with primary mouse anti-NNMT antibodies 1:200 (Santa Cruz) at 4°C overnight followed by the detection with goat anti-mouse Alexa Fluor 488 secondary antibodies (Thermo Fisher Scientific) at the 1:1000 dilution for 1 h at RT. Cells were washed three times with PBS, counterstained with DAPI and imaged using an inverted fluorescent IX51 microscope (Olympus, Valley, PA).

### Immunohistochemistry

Tissue Microarray (TMA) consisting of paraffin embedded tissue sections were de-paraffinized and rehydrated as follows: two incubations in xylene for 5 min each, followed by three incubations in 100% ethanol for 3 min each with subsequent serial dilutions of ethanol (95%, 70%, 50% and 20%) for 3 min each. After washing with H_2_O, the TMA sections were treated with Antigen Unmasking Solution (Vector Labs, Burlingame, CA) according to the manufacturer's recommendations, and then washed in PBS followed by treatment with 0.3% H_2_O_2_ in methanol for 30 min. After washing with PBS, TMA sections were blocked with normal goat serum for 20 min, and then incubated with primary mouse anti-NNMT antibodies (Santa Cruz) at the 1:100 dilution for 30 min, washed in PBS and incubated with biotinylated secondary anti-mouse antibodies (Vector Labs) for 30 min. Finally, the antigen-antibody complexes were detected with the Vectastain ABC Kit (Vector Labs), stained with ImmPACT/DAB staining solution (Vector Labs) for 30 min and counterstained with hematoxylin. Slides were dehydrated, air-dried and mounted with Permount (Thermo Fisher Scientific). All patients provided written informed consent for tissue storage and research use of donated cancer sample.

### Microarray-based comparative genomic hybridization

Genomic DNA was extracted using the DNeasy Tissue Kit as per manufacturer's instructions (Qiagen). Infinium CytoSNP-850k Beadchip array (Illumina, Essex, UK) was used according to the manufacturer's protocols to determine genome-wide copy number changes in OVCAR3 control, Gluc-2 and Gluc-3 cells. CytoSNP-850K BeadChip oligonucleotide arrays (Illumina) contains approximately 850,000 probes (50-mer long) with enriched coverage for 3262 genes of known relevance in cancer applications. Arrays were imaged using Illumina iScan and copy number values were determined by the Illumina BeadStudio software. All copy number calls are provided in Supplementary Table 2 (0: deletion/no call, 1: 1 copy, 2: 2 copies, 3: 3 copies (genomic gain), 4: 4+ copies (amplification)). Raw data files are available upon request.

### RNA sequencing

RNA extraction and quality control: RNA was extracted using RNeasy Mini Kit (Qiagen, Valencia, CA) and quantified using NanoDrop 8000 Spectrophotometer (Thermo Scientific, Carlsbad, CA). RNA was qualified using the Agilent RNA 6000 Nano Kit and the Agilent Bioanalyzer (Agilent Technologies, Santa Clara, CA), only high quality samples with high RNA integrity numbers (RIN < 9) were used. Biological replicates of OVCAR3 control, OVCAR3 Gluc-2 and OVCAR3 Gluc-3 cells were performed to enable downstream statistics.

Library preparation and sequencing: 1 μg of total RNA from high quality samples was used as an input into the Ion Total RNAseq kit v2 (Ion Torrent, Carlsbad, CA), in which sample RNA was fragmented and reverse transcribed, adapter-ligated and amplified according to manufacturer's instructions. Sequencing adapters were ligated from the Ion Xpress^™^ Plus Fragment Library Kit (Life Technologies). Libraries were quantified with Qubit (Invitrogen) and qualified with the Agilent Bioanalyzer (Agilent Technologies, Santa Clara, CA) before being pooled in equimolar amounts and amplified onto Ion Sphere Particles using Ion PI^™^ template OT2 200 Kit v3 (Life Technologies). Finally, the samples were sequenced on the Ion Proton^™^ using semi-conductor sequencing with Ion PI™ Sequencing 200 v3 kit (Life Technologies) to a depth of 12 to 25 million reads with a mean read length of 90 bp.

Bioinformatics: In brief, raw reads were filtered and trimmed by FASTX toolkit (http://hannonlab.cshl.edu/fastx_toolkit/) then aligned to human reference genome (hg19) using TMAP with the Gencode version 19 reference human genome annotation (http://www.gencodegenes.org). FPKM (fragment per kilobase of gene per million reads sequenced) values were calculated for 23,847 genes using Cufflinks 2.0.8. Poorly measured raw FPKM values less than 1 were increased to a floor threshold of 1.1. Raw FPKM values were then log2 transformed. Genes with an average of 1 or less in addition to miRNAs and SNORDs were removed from further analysis. In general, libraries contained less than 2.5% of reads coming from ribosomal RNA and over 90% of reads mapping to the genome suggesting high quality RNA libraries for downstream analysis. A two-tailed t-test was used to assess the significance of gene expression differences and then corrected for multiple hypotheses by calculating the q value using the Benjamini and Hochberg method. Data from well-annotated genes that had significant gene expression differences with a false discovery rate below 20% (defined as a *q* value < 0.2) are presented.

### Biolog phenotype microarrays

Metabolic profiling was performed with Biolog Phenotype Microarrays (Biolog, Hayward, CA) according to manufacturer's recommendations. Briefly, cells were seeded in PM-M1 and PM-M2 96-well plates. The PM-M1 array consists of wells coated with carbohydrate and carboxylate substrates, whereas PM-M2 contains glutamine, individual L-amino acids and most dipeptide combinations. Cell lines were seeded into Biolog Microarray wells and cultured at 37°C, 5% CO_2_ in a humidified atmosphere and in Biolog IF-M1 proprietary medium without glucose, but with low levels of glutamine (0.3 mM), 5% FBS and 1% Penicillin/Streptomycin solution. The assays were incubated for 48 h and the colorimetric reaction was developed with MB dye for 6 h. Color intensity reflects the level of energy produced by cells as a measure of NAD^+^ reduction to NADH. Absorbance for all substrates was measured at 600 nm using Glomax Multi Detection System (Promega) and the values of absorbance were normalized by deducting the negative control absorbance (uncoated wells) from the absorbance value of coated wells, and dividing it by the average of positive control (wells coated with α-D-glucose). Statistical calculations were performed using a two-tailed Student's *t*-test (*P < 0.05) by comparing normalized absorbance values for a given substrate measured in two independent biological and technical replicates with the readings for another cell line. Biolog Phenotype Microarrays were performed on glucose-restricted OVCAR3 sublines (Gluc-2 and Gluc- 3), control OVCAR3, OVCAR3-GFP and -ZEB1 cells, OVCA433-GFP and -ZEB1 cells, as well as OVCAR3 Gluc-2 and Gluc-3 sublines transformed with shCtrl and shNNMT-3.

### Computational analyses

### Kaplan-Meier plots

The Kaplan-Meier plotter (http://kmplot.com) [60] was used to generate Kaplan-Meier plots of overall (OS) and progression-free survival (PFS) of ovarian cancer patients from TCGA dataset, expressing high and low levels of NNMT. The criteria for generation of plots were set up to analyze only patients with ovarian cancer of high-grade serous histology (stage III and IV).

### Classification to different HGSC subtypes

NNMT expression in different HGSC subtypes was analyzed using R2 Genomics Analysis and Visualization Platform (R2: Genomics Analysis and Visualization Platform (http://r2.amc.nl)). ‘Tumor Ovarian Serous Cystoadenocarcinoma dataset’ (TCGA, 541 patients) and ‘Ovarian Tumor’ dataset (Tothill *et al*., 285 patients [33]) were used to generate graphs.

### Gene Ontology analyses

Gene Ontology enrichment analysis of RNAseq results was performed using DAVID Bioinformatics Resources 6.7 [74].

### GSEA analyses

Gene Set Enrichment Analysis GSEA software (the Broad Institute) was used to determine the overlap of transcriptional changes observed in glucose-restricted sublines with published hallmark signatures and other defined Gene Set Databases, as well as to create graphs visualizing Enrichment Maps [75].

### Expression of NNMT in normal tissues and corresponding cancers

Box plots for differential NNMT expression were generated using datasets (U133Plus2 platform: 17931 cancers and 3503 normal tissues) and software available through the Gene Expression across Normal and Tumor tissue (GENT) portal (medical-genome.kribb.re.kr/GENT) [76]. Average NNMT expression levels across all analyzed normal (N) or cancer (C) tissues of different origin are indicated by vertical dotted green and red lines, respectively.

### Statistical analysis

Data were analyzed using a Student's *t*-test, two-tailed distribution with two-sample equal variance (homoscedastic), calculated in GraphPad Prism version 6 software (San Diego, CA).

## SUPPLEMENTARY MATERIALS FIGURES AND TABLES














