# Supplementary material for: Glucose deprivation elicits phenotypic plasticity via ZEB1-mediated expression of NNMT
Source: Oncotarget. 2017 Feb 17;8(16):26200–20. doi: 10.18632/oncotarget.15429 (PMC5432250; doi:10.18632/oncotarget.15429)
Supplement: Supplementary file 1 [file oncotarget-08-26200-s001.pdf]

Glucose deprivation elicits phenotypic plasticity via ZEB1-mediated expression of NNMT

Supplementary Materials

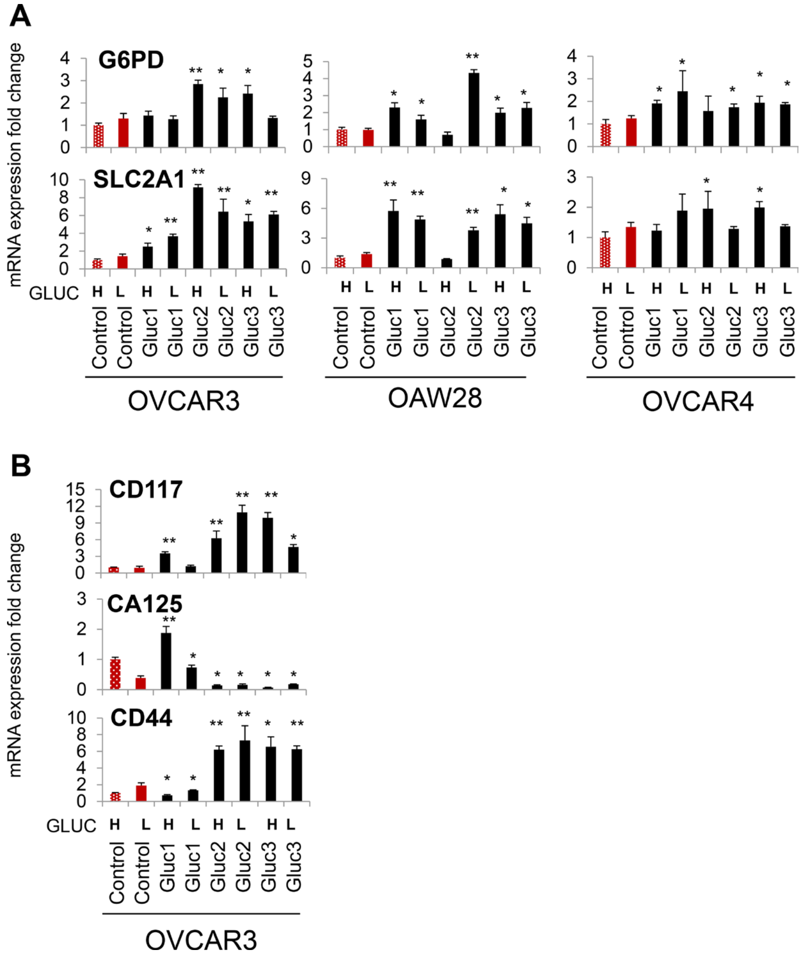

Supplementary Figure 1: Glucose-restricted sublines derived from OVCAR3, OVCAR4 and OAW28 cell lines elevate expression of metabolic regulators (G6PD and SLC2A1) and ovarian cancer stem cell markers (CD44 and CD117). (**A**) Glucose-restricted OVCAR3, OVCAR4 and OAW28 sublines demonstrate increased expression of metabolic genes, *G6PD* and *SLC2A1,* compared to their control cells. (**B**) Glucose-restricted OVCAR3 sublines acquire gene expression changes associated with ovarian cancer stem cells (CSC), such as elevated *CD117, CD44* and decreased *CA125 (MUC16)*. For all figure panels, statistical calculations were performed using a two-tailed Student's *t*-test (* $0.001 < P < 0.05$; ** $P < 0.001$). Red bars represent transcriptional changes in control cells and black bars represent relative mRNA expression in glucose-restricted cells. Control cells were cultured in low glucose (L) DMEM for 2 d, while glucose-restricted cells were maintained in low glucose and exposed to DMEM with high glucose (H) for 7 d prior to harvesting cells for analyses of transcriptional changes. All cells were cultured in normal-seeding density conditions and harvested at confluency lower than 80%.

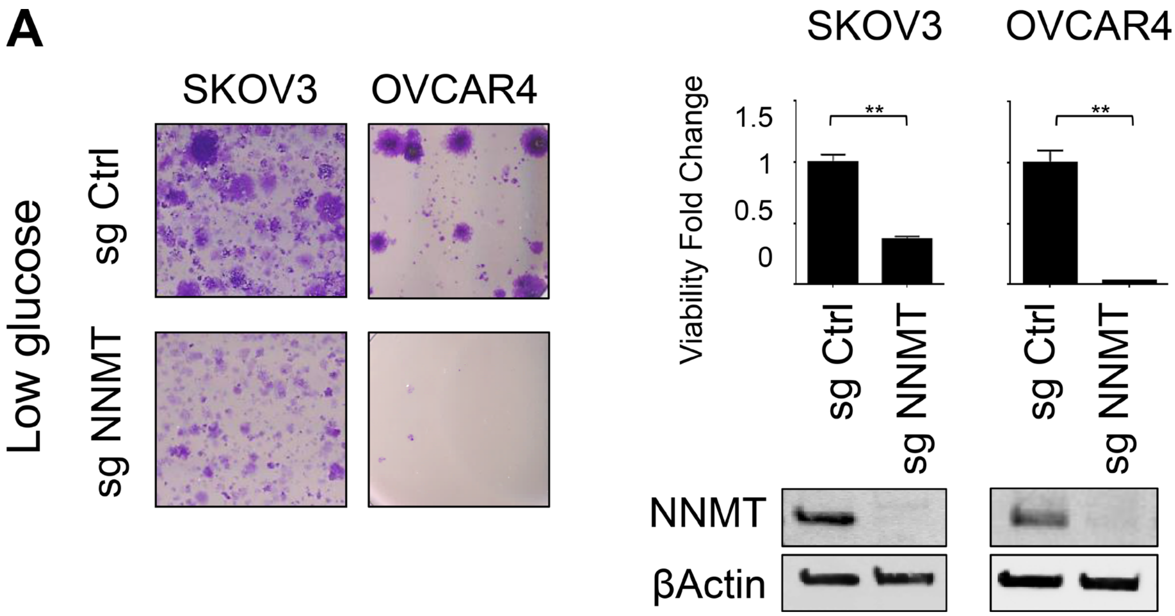

Supplementary Figure 2: *NNMT* depletion reduces viability of parental OVCAR4 and SKOV3 cells in low glucose conditions. (A) CRISPR/Cas9-mediated *NNMT* depletion in parental ovarian cancer cell lines with high baseline NNMT expression, such as SKOV3 and OVCAR4, caused drastic reduction in the total number of viable cells upon culturing in low seeding density conditions in low glucose levels. Graph shows fold change of absorbance measured after extracting crystal violet from control and NNMT-depleted cells after 14 d. Statistical calculations were performed using a two-tailed Student's *t*-test (* $0.001 < P < 0.05$; ** $P < 0.001$).

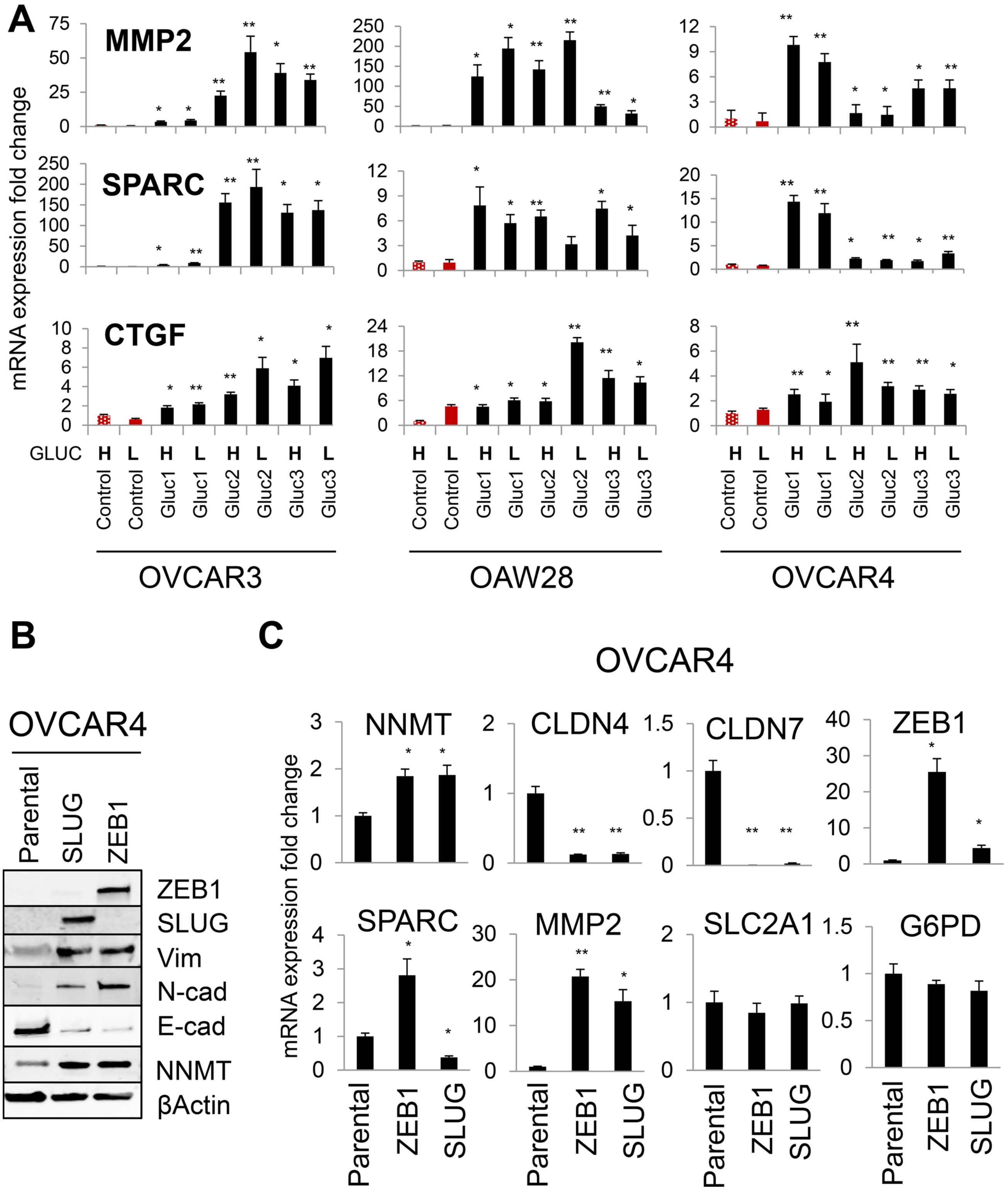

Supplementary Figure 3: Glucose-restricted sublines derived from OVCAR3, OVCAR4 and OAW28 cell lines commonly upregulate expression of mesenchymal regulators and ZEB1 overexpression recapitulates these mesenchymal-like gene expression changes. (**A**) mRNA expression of *SPARC, CTGF* and *MMP2* is consistently upregulated in all glucose-restricted sublines generated from OVCAR3, OVCAR4 and OAW28 cell lines. Red bars represent transcriptional changes in control cells and black bars represent relative mRNA expression in glucose-restricted cells. Cells were cultured as described in Supplemental Figure 1B. (**B**) *ZEB1* and *SLUG* overexpression in OVCAR4 cells induced epithelial-to-mesenchymal transition as manifested by elevated Vimentin, N-cadherin and NNMT protein levels, as well as decreased E-cadherin expression. (**C**) Ectopic *ZEB1* expression in OVCAR4 cells increased *NNMT, MMP2, SPARC* and decreased *CLDN4* and *CLDN7* expression, but had no effect on the *SLC2A1* and *G6PD* expression. For all figure panels, statistical calculations were performed using a two-tailed Student's *t*-test (* $0.001 < P < 0.05$; ** $P < 0.001$).

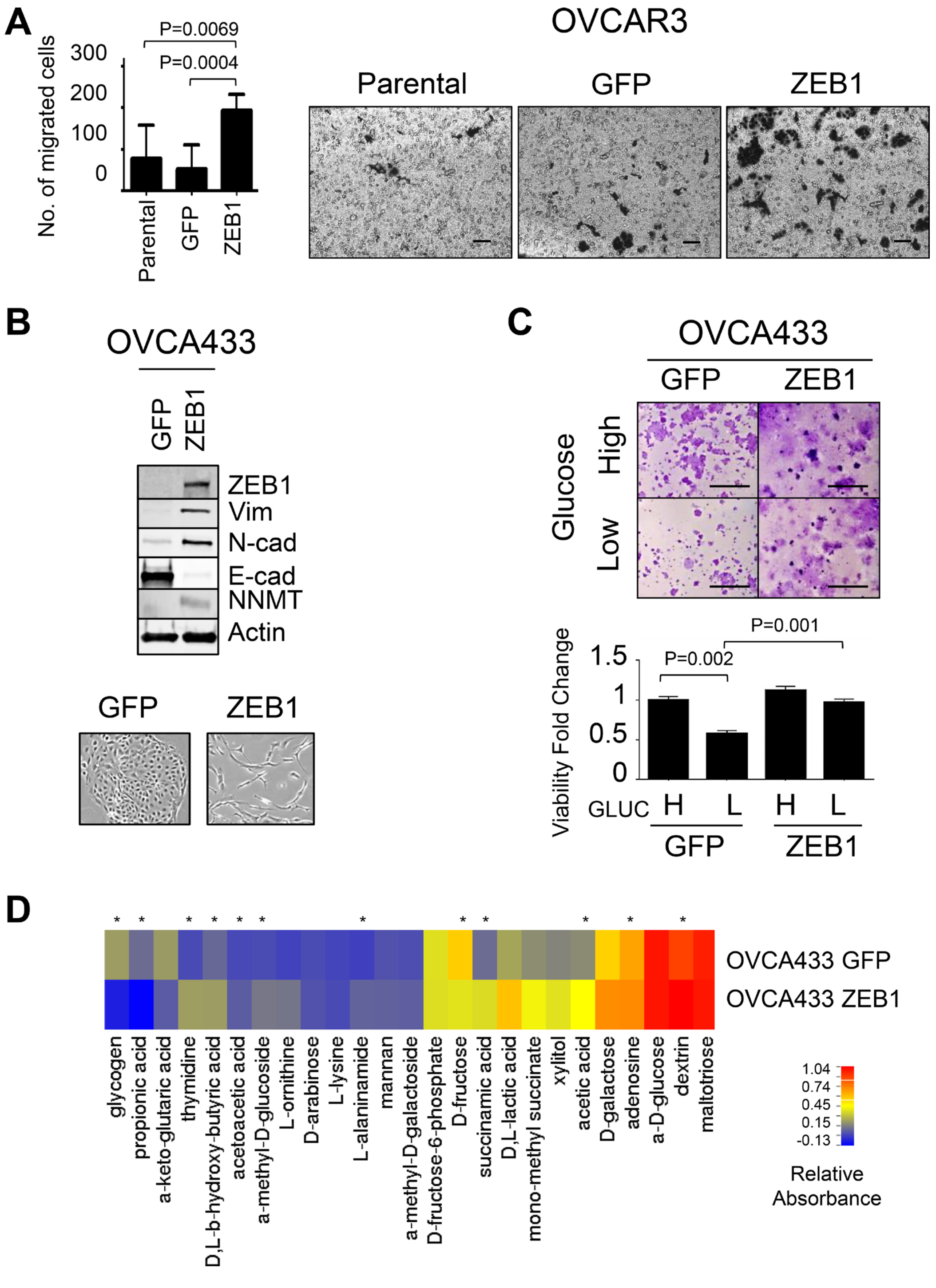

Supplementary Figure 4: ZEB1 overexpression in OVCAR3 and OVCA433 cells recapitulates glucose resistance. (**A**) Overexpression of *ZEB1* in OVCAR3 cells significantly increased their migratory potential compared to parental or GFP-expressing cells. Scale bar: 100 µm. (**B**) OVCA433 ovarian cancer cells ectopically expressing *ZEB1* undergo expression changes reminiscent of epithelial-to-mesenchymal transition (EMT): decreased expression of E-cadherin and increased expression of N-cadherin, Vimentin and NNMT. (**C**) Ectopic *ZEB1* expression increased cellular viability of OVCA433 cells upon glucose deprivation. Graph shows total number of viable cells determined by staining cells with crystal violet after 14 d of culture. Scale bar: 5 mm. (**D**) Ectopic *ZEB1* expression recapitulates metabolic adaptations observed in glucose-restricted cells, such as increased utilization of sugars (D-galactose, dextrin, xylitol, mannan and D-arabinose), ketones (D, L-β-hydroxy-butyric acid and acetoacetic acid), D, L-lactic acid, methylated substrates (α-methyl-D-galactoside, α-methyl-D-glucoside and mono-methyl succinate) in the absence of glucose. Asterisks (*) denote substrates differentially utilized between control and transformed cells ($P < 0.05$). Statistical calculations were performed using a two-tailed Student's *t*-test (* $0.001 < P < 0.05$; ** $P < 0.001$).

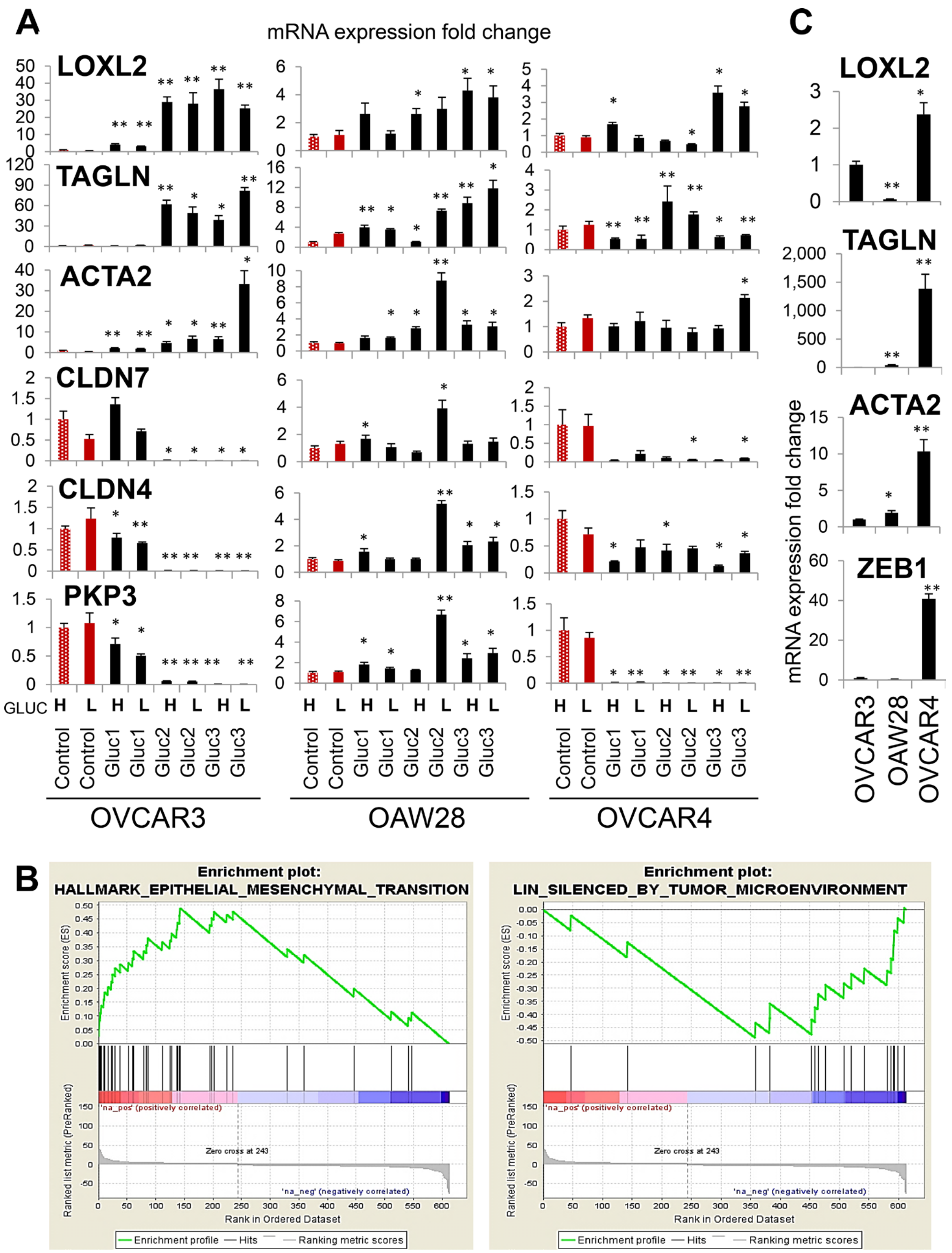

Supplementary Figure 5: Glucose-restricted sublines derived from OVCAR3, OVCAR4 and OAW28 cell lines upregulate mesenchymal gene expression and weaken cell-cell interactions. (**A**) Glucose-restricted OVCAR3 sublines show elevated expression of mesenchymal genes, such as *LOXL2, TAGLN* and *ACTA2*. Mesenchymal gene expression is highest in OVCAR3 Gluc-2 and Gluc-3 cells, which demonstrate more prominent EMT features. Glucose-restricted OVCAR3 sublines also show decreased expression of genes encoding for integral membrane proteins that mediate interactions between neighboring cells in desmosomes and tight junctions (*CLDN4, CLDN7* and *PKP3*). Glucose-restricted OVCAR4 and OAW28 cells acquire mesenchymal-like gene expression changes; however, they do not show classic EMT gene expression. While glucose-restricted OVCAR4 sublines lose expression of genes coding for cell-cell interaction proteins, such as *CLDN4, CLDN7* and *PKP3*, all OAW28-Gluc sublines retain expression of genes involved in cell-cell junctions. In contrast, OAW28-Gluc sublines upregulate *LOXL2, TAGLN* and *ACTA2* expression, while OVCAR4 sublines do not consistently alter expression of those mesenchymal genes. Red bars represent transcriptional changes in control cells and black bars represent relative mRNA expression changes in glucose-restricted cells. Cells were cultured as described in Supplemental Figure 1B. (**B**) GSEA analysis of genes upregulated in the OVCAR3 Gluc-2 and Gluc-3 sublines show statistically significant enrichment for genes involved in EMT, whereas downregulated genes were enriched for genes silenced by co-culture with cancer associated fibroblasts (CAFs). (**C**) Parental OVCAR4 cells intrinsically demonstrate higher expression of mesenchymal genes, such as *LOXL2, TAGLN, ACTA2* and *ZEB1* compared to OVCAR3 and OAW28 cell lines. For all figure panels, statistical calculations were performed using a two-tailed Student's *t*-test (* $0.001 < P < 0.05$; ** $P < 0.001$).

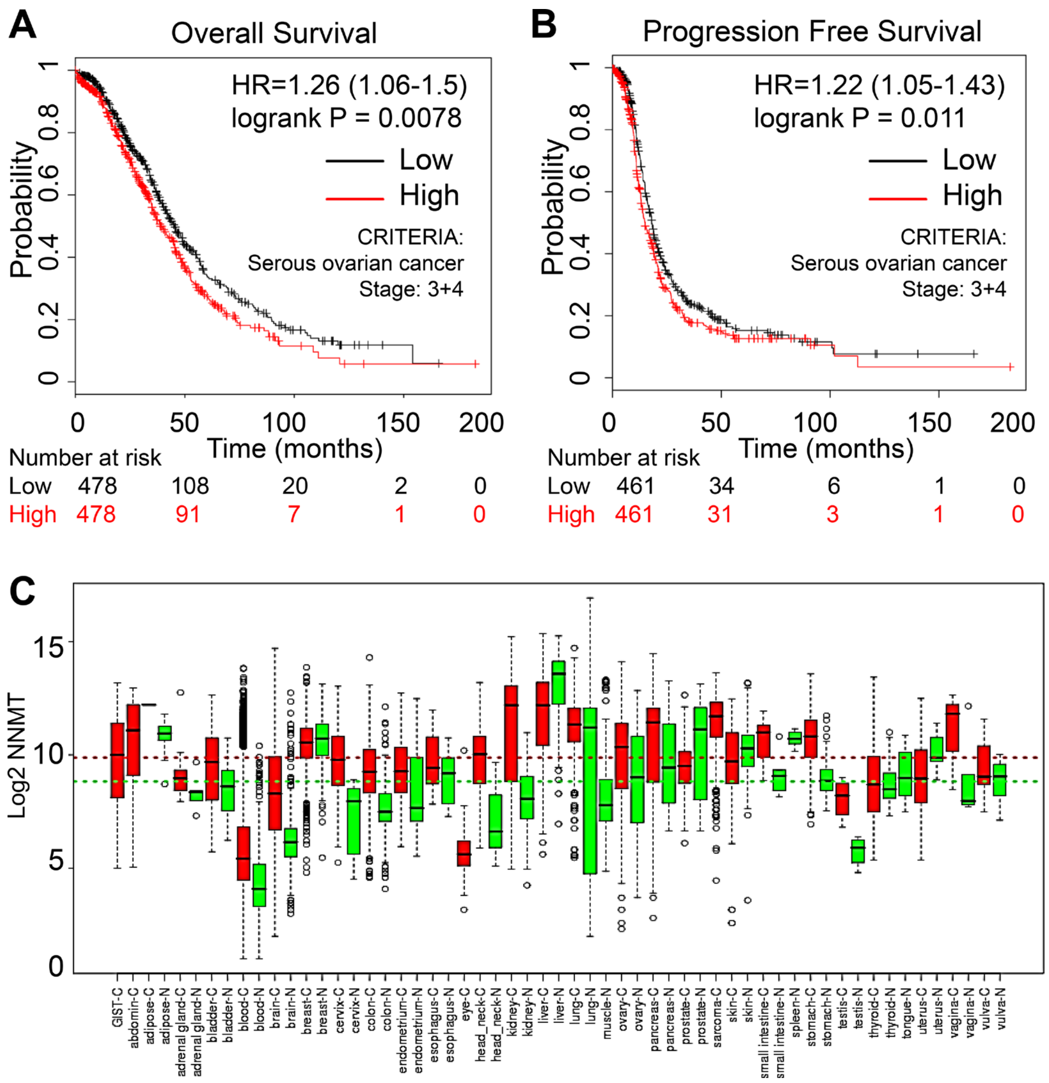

Supplementary Figure 6: High *NNMT* expression is associated with worse overall and progression free survival in HGSC. (**A**) Kaplan-Meier Plot shows that high *NNMT* expression in patients with stage III and IV high-grade serous ovarian carcinomas (n = 956) is significantly correlated with shorter overall survival (OS, P = 0.0078). (**B**) Kaplan-Meier Plot showing shorter progression free survival (PFS) in patients with stage III and IV high grade serous carcinomas (n = 922) with high *NNMT* expression compared to patients with low *NNMT* expression (P = 0.011). (**C**) *NNMT* is differentially expressed between cancers and corresponding normal tissues. Comparison of *NNMT* expression in 17931 cancers and 3503 normal tissues revealed that *NNMT* mRNA is elevated in most cancers (C) in comparison to their corresponding normal tissues (N). Average NNMT expression levels across all analyzed normal or cancer tissues of different origin are indicated by vertical dashed green and red lines, respectively. The y axis shows log2 *NNMT* mRNA levels. Consistently, *NNMT* levels in ovarian cancer are higher than in normal ovary.

Supplementary Table 1: List of significantly altered genes in OVCAR3 Gluc-2 and Gluc-3 compared to control cells. The fold change (FC) represents the mean of fold change of FPKM values determined by RNA sequencing ($P < 0.05$, $q < 0.2$). See Supplementary_Table_1

Supplementary Table 2: Copy number values determined by array-CGH for control OVCAR3, OVCAR3-Gluc2 and OVCAR3-Gluc3 (0: deletion, 1: one copy (loss), 2: two copies (diploid), 3: three copies (gain), 4: 4+ copies (amplification)). See Supplementary_Table_2

Supplementary Table 3: Biological processes altered in OVCAR3 Gluc sublines identified by Gene Ontology enrichment analysis. See Supplementary_Table_3

Supplementary Table 4: The list of published gene signatures and hallmarks, which show statistically significant enrichment in OVCAR3 Gluc-2 and Gluc-3 sublines compared to control OVCAR3 cells ($P < 0.05$). The analyses were performed using Gene Set Enrichment Analyses (GSEA) software and database. See Supplementary_Table_4

Supplementary Table 5: List of genes commonly expressed between mesenchymal subtype of HGSC (TCGA), the C1 (Tothill *et al.*) subtype of HGSC and the glucose withdrawal signature. Glucose withdrawal signature represents genes commonly upregulated ($P < 0.05$) upon chronic glucose withdrawal in OVCAR3 Gluc-2 and Gluc-3 compared to control OVCAR3 cells. See Supplementary_Table_5

Supplementary Table 6: List of primers used for the quantitative reverse transcriptase (qRT-PCR) and genomic PCR (genomic qPCR) reactions. See Supplementary_Table_6
